# COVID-19対策と日本国憲法：新型インフルエンザ等対策特別措置法に着目して

**DOI:** 10.12688/f1000research.50861.1

**Published:** 2021-03-23

**Authors:** Hajime Akiyama

**Affiliations:** 1 Faculty of Humanities and Social Sciences, University of Tsukuba, Tsukuba, Ibaraki, 305-8571, Japan

**Keywords:** 新型コロナウイルス感染症, 日本国憲法, 新型インフルエンザ等対策特別措置法, 人権, 営業の自由, 移動の自由, 生命権, 生存権, 公衆衛生, 公共の福祉, COVID-19, the Constitution of Japan, the Act on Special Measures for Pandemic Influenza and New Infectious Diseases Preparedness and Response, human rights, freedom of establishment, freedom of movement, right to life, welfare rights, public health, public welfare

## Abstract

2020年3月以来、日本においては新型インフルエンザ等対策特別措置法がCOVID-19対策の中心的な役割を果たしてきた。同法に基づき、休業や営業時間短縮の要請・指示、外出自粛要請が行われている。2021年1月時点で、罰則はないものの、これらの要請・指示には日本国憲法が保障する営業の自由や移動の自由を制限する側面がある。そこで本稿は、「COVID-19対策としての休業や営業時間短縮の要請・指示及び外出自粛要請に、憲法上の制約もしくは要請はあるか」をリサーチ・クエスチョンとして検討を行った。また、「罰則のある措置は憲法上認められるか」との論点も扱った。本稿は「個人の自由を保障する概念」及び「個人の自由を制限しうる概念」に分けて検討した。個人の自由を保障する概念としては、営業の自由と移動の自由が挙げられる。これらの自由は主に居住、移転及び職業選択の自由（憲法22条）及び財産権（同29条）により保障される。また、生命権（同13条）、生存権・公衆衛生（同25条）及び公共の福祉（同13条）への脅威となる個人の自由は制限されうる。本稿は、COVID-19対策が公共の福祉に適合するため、憲法は営業の自由及び移動の自由の制限を許容していると主張した。また、生命権、生存権・公衆衛生の視点から、政府がCOVID-19に起因する生命へのリスクを低減させる責任を負っていると述べた。さらに比例原則を検討する必要はあるものの、憲法は罰則のある措置を許容していると論じた。

## Abstract (en)

Since March 2020, the Act on Special Measures for Pandemic Influenza and New Infectious Diseases Preparedness and Response has been a significant statute in dealing with COVID-19 in Japan. The Act mandates requests and orders for business suspension and shortened business hours, as well as stay-at-home requests. Although there have been no penalties as of January 2021, these requests and orders limit freedom of movement and establishment, guaranteed rights under the Japanese Constitution. This article poses the following research question: “Does the Japanese Constitution allow measures against COVID-19 such as requests and orders for business suspension and shortened business hours, and stay-at-home requests?” It also asks: “Are measures with penalties allowed by the Constitution?” This paper introduces constitutional concepts that guarantee or limit individual freedom. Concepts that guarantee individual freedoms include freedom of establishment and movement. These freedoms derive from the constitutional values of freedom to choose one’s occupation and choose and change one’s residence (Art. 22) and the right to own or hold property (Art. 29). Concepts that limit individual freedom include the right to life (Art. 13), welfare rights and public health (Art. 25), and public welfare (Art. 13). Individual freedom that threatens right to life, welfare rights and public health, and public welfare may not be guaranteed. This paper argues that since measures against COVID-19 are considered public welfare, the Constitution allows the limiting of freedom of establishment and movement. Furthermore, from the perspectives of the right to life, welfare rights, and public health, the government is responsible for reducing the risk to life from COVID-19. It also argues that the Constitution permits measures with penalties, while proportionality needs to be considered.

## はじめに

2020 年から 2021 年にかけて、世界各国で新型コロナウイルス感染症 （以下、COVID-19） の感染確認者数が増加している。2021 年 1 月の時点で、日本を含め多くの国では COVID-19 のワクチンがまだ承認されておらず、今後確保できるワクチン数、変異したウイルスへのワクチンの効力等は不明である。そこで各国政府は感染拡大予防のために、様々な対策を採っている。例えば各国の COVID-19 対策を分析している Our World in Data によれば、2020 年 12 月 31 日現在、124 の国や地域で外出自粛要請や外出制限などの措置が取られている ^1^。

日本においては、2020 年 3 月以来「新型インフルエンザ等対策特別措置法 （以下、特措法） 」が COVID-19 対策の中心的な役割を果たしてきた。同法に基づき、休業や営業時間短縮の要請・指示、外出自粛要請が行われてきた。2021 年 1 月時点で、罰則はないものの、これらの要請・指示には日本国憲法が保障する営業の自由や移動の自由を制限する側面がある。その一方で、COVID-19 対策が憲法に規定される公共の福祉に適合しており、自由の制限はやむを得ないとの見解もあり得る。

2021 年 1 月現在、日本の COVID-19 対策の憲法上の論点について包括的に検討した論文は限られている ^2^。その一つが、江藤祥平による論文である ^3^。江藤は「公共の福祉」と「個人の人権」という図式を提示し、感染症対策のための私権の制限の合憲性について詳細かつ批判的に検討している。同論文は憲法における公衆衛生、表現の自由、営業の自由、個人の尊厳、自己決定権にとどまらず、国際人権法における健康への権利にも言及しており、多角的に COVID-19 対策について検討を行っている。しかし、江藤論文における分析に加えて、以下の二点の検討が必要である。第一に、憲法 13 条に規定される生命権を検討する必要がある。通説では生命権について議論されてこなかったが、COVID-19 の感染拡大は人権の基盤である生命への脅威となり、生命権の概念を含めて COVID-19 対策を検討し直す必要がある。第二に、「公共の福祉」と「個人の人権」という分類の妥当性を再検討する必要がある。江藤の述べる通り、この二項対立は憲法学では「お馴染みの図式」である ^4^。しかし COVID-19 対策においては、公共の福祉と人権でなく、人権規範同士が衝突することも想定される。例えば COVID-19 が生命権への脅威であるとすれば、感染拡大予防策により生命権の保障が見込まれる一方で、江藤が「個人の人権」とする自由権的な権利が制限される可能性がある。すなわち、「公共の福祉」と「個人の人権」でなく、権利の性質を考慮しつつ、「個人の自由を保障する概念」及び「個人の自由を制限しうる概念」に整理し直し、COVID-19 対策の憲法上の論点を再検討する必要性がある。

本稿のリサーチ・クエスチョンは、「COVID-19 対策としての休業や営業時間短縮の要請・指示及び外出自粛要請に、憲法上の制約もしくは要請はあるか」である。また、COVID-19 の感染拡大により、罰則のある措置を盛り込んだ特措法の改正が議論されている 2021 年 1 月現在の状況を踏まえ ^5^、「罰則のある措置は憲法上認められるか」との論点も検討する ^6^。

なお、本稿は特措法に基づく対応に着目して検討する。特措法以外にも、感染症法上の指定感染症 ^7^、検疫法上の措置、予防接種法上のワクチン等の論点があるが、これらは別稿に譲りたい。その上で、本稿は特措法に基づく対策の総論的な論点の整理に留め、各論的な議論の詳細には立ち入らない。例えば首長による要請・指示については、行政法の視点を導入する必要があるが、論点が散逸するため詳細には扱わない。また、本稿は 2021 年 1 月 10 日以前の情報を基盤としており、COVID-19 の状況、政府の対応は刻一刻と変化することにご留意いただきたい。

第 1 節では、特措法に基づき採られた COVID-19 対策を紹介する。第 2 ・ 3 節は、COVID-19 対策に関連する、個人の自由を保障する概念及び個人の自由を制限しうる概念を指摘する。第 4 節では、上記の概念を COVID-19 対策に適用し、COVID-19 対策を検討する際の憲法的論点を整理する。

## I. 特措法と COVID-19 対策

2021 年 1 月現在、日本における COVID-19 対策の基盤となっているのは、特措法である。特措法は、2009 年に新型インフルエンザが世界的に流行し、医療資源が逼迫したことを契機として、2012 年に成立し、2013 年に施行された ^8^。特措法の対象は新型インフルエンザ、再興型インフルエンザ及び新感染症である ^9^。ここで重要なのは、特措法の対象となる感染症は、国民の多くが免疫を獲得していないため、急速な蔓延により国民の生命及び健康に重大な影響を与える恐れがある点である ^10^。そのために、既知の感染症より強い措置が必要となる。

当初、特措法は COVID-19 に適用されず、感染症法及び検疫法を基盤とした対応が想定されていた ^11^。特措法の特徴は、緊急事態宣言や外出自粛要請、施設の使用制限要請・指示が可能になることであるが、厚生労働省幹部によれば、日本で初めて COVID-19 の感染者が確認された 2020 年 1 月時点では特措法の適用が必要であるとの認識は共有されていなかったようである ^12^。同年 2 月 1 日に、COVID-19 は感染症法に基づく二類相当の「指定感染症」とされ、感染の疑いがある患者の入院措置が可能になった ^13^。また同日、COVID-19 が検疫法上の「検疫感染症」となり、強制力を伴う入国時の診察・検査が可能になった ^14^。

しかし同年 2 月 21 日に国内感染者数が 100 人を超えると社会的な緊張が高まり、2 月 27 日には当時の安倍首相が、感染拡大抑制のために必要な法案を早急に準備するよう指示した ^15^。そして 3 月 13 日に特措法が改正され、翌 14 日より COVID-19 が特措法の対象となった ^16^。

特措法は二段階構造により、新型感染症のまん延予防を試みている ^17^。第一段階は政府対策本部の設置である。従来のインフルエンザより症状が深刻である新型感染症が発生した際に、政府対策本部、都道府県対策本部が設置される ^18^。都道府県対策本部長となる都道府県知事は、特措法 24 条 9 項に基づき、団体、個人に必要な協力を要請する事ができる。

第二段階が緊急事態宣言である。首相は、感染症が全国的なまん延により、かつ国民社会、国民生活に甚大な影響を及ぼす場合に緊急事態宣言を発出し、緊急事態措置を実施すべき期間、区域を指定する ^19^。特措法は緊急事態宣言発出時の専門家の役割に触れていないが、首相が専門家に諮問することが想定されている ^20^。緊急事態措置の対象となった地域では、都道府県知事が不要不急の外出自粛要請及び施設使用制限の要請を行う事ができる ^21^。施設の使用制限要請に応じない場合、知事は施設管理者に法的義務を課す指示を行うことができる ^22^。これらの要請・指示に従わなくとも罰則はない。しかし、45 条に基づく施設使用制限が要請・指示された場合は、その旨が公表される ^23^。公表が可能になることが、緊急事態宣言発出による大きな変化である。しかし、政府対策本部が設置されていれば都道府県知事は必要な協力を要請できるため、緊急事態宣言の発出は社会的に大きなインパクトがあるものの、要請の法的な効力が変わるわけではないことには留意が必要である ^24^。

緊急事態措置の対象となった地域では、都道府県知事が要請・指示の主体となるが、政府が定める基本的対処方針 （特措法 18 条） に沿って要請・指示を行うこととなる ^25^。また、新感染症対策であっても、国民の自由や権利への制限は最低限であるべきであると規定されている ^26^。

2020 年 3 月 14 日に COVID-19 が特措法の対象となると、同法 15 条に基づく政府対策本部が設置され ^27^、各都道府県知事が同法 24 条に依拠して、団体・個人への要請が可能になった。同月には大阪府や東京都、神奈川県、埼玉県、千葉県、山梨県等が外出自粛を要請した ^28^。感染確認者数の増加と社会的危機感の高まりを受け ^29^、2020 年 4 月 7 日から 5 月 25 日にかけて緊急事態宣言が発出された ^30^。緊急事態措置の対象となった都道府県では特措法 45 条に基づく不要不急の外出自粛要請、施設使用制限の要請・指示が可能になった。実際に全ての都道府県で同法 45 条 1 項に基づいた不要不急の外出自粛が要請され、21 都道府県で同法 45 条 2 項に基づき、休業や営業時間短縮要請をはじめとする施設の使用制限が要請され、施設管理者が公表された ^31^。また、5 県ではさらに同法 45 条 3 項に基づく指示及び同法 45 条 4 項に基づく公表が行われた ^32^。緊急事態宣言解除後も、同法 24 条 9 項を根拠として、断続的に外出自粛要請を行った都道府県がある ^33^。また、2020 年 4 月の緊急事態宣言発出後は、解除後も含め、散発的に同法 24 条 9 項を根拠とした休業や営業時間短縮の要請を出している都道府県もある ^34^。また、2020 年末から感染確認者数が 1 日に 3000 人を超える日が続き、重症者数も多いため ^35^、2021 年 1 月 7 日に、1 月 8 日から 2 月 7 日を期間とする緊急事態宣言が発出された ^36^。東京都、神奈川県、千葉県、埼玉県が緊急事態措置の対象とされている ^37^。

## II. 個人の自由を保障する概念

罰則を伴わないとはいえ、特措法による休業や営業時間短縮要請・指示、外出自粛要請は、国家権力による市民の自由を制限する要請・指示である。国家による介入の回避を重要視する近代立憲主義を基盤とし ^38^、また大日本帝国憲法下での国家権力の強大化を経験した後に制定された日本国憲法の視点からは、市民の自由の制限が重要な論点となりうる。本節では、日本国憲法が保障する個人の自由として、営業の自由及び移動の自由についての判例や学説を検討する。

休業や営業時間短縮の要請・指示は、営業の自由を制限しうる。営業の自由に関連する条文は、憲法 22 条、29 条、13 条である。営業の自由自体を保障する文言は憲法に存在しないが、判例、通説によれば憲法 22 条の職業選択の自由により営業の自由が保障されると考えられている ^39^。また、 22 条及び 29 条に規定される財産権の双方により営業の自由が保障されるとの見解もある ^40^。その上で、憲法 13 条に規定される人格権と関連して営業の自由を位置付けることもできる。薬事法違憲判決は、職業が「個人の人格的価値とも不可分の関連を有する」としており ^41^、学説でも営業の自由と人格的価値の関連が指摘されている ^42^。人格的価値は憲法 13 条から導出できる人格権と関連しており ^43^、営業の自由が人格権の視点からも重要であることを示唆している。

外出自粛要請は、移動の自由を制限しうる。関連する条文は憲法 22 条及び 13 条である。移動の自由は、居住・移転の自由を規定する憲法 22 条に基づき保障されると考えられている ^44^。その上で、移動の自由は人の活動領域を拡大し、人格形成に寄与するとの指摘もあり ^45^、憲法 13 条を基盤とする人格権として移動の自由が捉えられる可能性もある。

## III. 個人の自由を制限しうる概念

憲法には個人の自由を保障する概念がある一方で、個人の自由を制限しうる概念も存在する。生命権、生存権・公衆衛生、公共の福祉の脅威となる場合、自由は制限されうる。

第一に、生命権の脅威となる自由は制限されうる。生命権の根拠は憲法 13 条である。日本国憲法における生命権は、1987 年に櫻田が提唱し ^46^、石村 ^47^、ウィリアムズ ^48^、小林 ^49^が議論を深め、山内が 2000 年に体系化を試みた ^50^。しかし通説では、憲法 13 条の「生命、自由及び幸福追求に対する国民の権利」が幸福追求権を意味するとされ ^51^、判例も憲法 13 条が生命権を規定するとの理解を示しておらず ^52^、生命権については十分議論されてこなかった ^53^。しかし、生命権は以下の二つの理由から、幸福追求権及び人格権とは別個に理解されるべきである。第一に、条文の文理上の解釈である。生命に対する権利、自由に対する権利及び幸福追求に対する権利は並列で表記されているため、生命に対する権利が幸福追求に対する権利に包含されるべきではない ^54^。第二に、権利の性質である。生命の維持は幸福追求権を含むあらゆる権利の前提であり、「もっとも基本的な人権」と考えられるべきである ^55^。生命への権利は、国王の圧政からの解放という近代憲法成立の文脈では、「国家に殺されない権利」が中心的な議論であり、生命権においても主に国家の介入を否定する自由権的な文脈で議論されてきた ^56^。しかし、COVID-19 への対応が必要となる今日においては、国家の積極的な介入を要求する社会権的な視点でも生命権をとらえる必要があり、国家による生命の保護義務も含まれるべきである ^57^。COVID-19 による死者は 2021 年 1 月 10 日現在、日本で 4043 人確認されており ^58^、COVID-19 が生命権の脅威となることを示している ^59^。よって、生命権保障の手段として COVID-19 対策を捉えることができる。

第二に、憲法 25 条の生存権の保障・公衆衛生の保全を目的とした自由の制限は認められうる。日本では 2021 年 1 月時点で、COVID-19 に有効なワクチンは未承認であり、また 2020 年終盤から確認されている変異したウイルスへのワクチンの効果も明らかでない ^60^。よって現状では、「健康で文化的な最低限度の生活を営む権利を有する」とする憲法 25 条を根拠として、ワクチン接種以外の感染症対策が必要とされよう。通説、判例によれば、憲法 25 条に基づく具体的な請求権は認められていない ^61^。従来、憲法 25 条と請求権については、生活保護などの給付請求権について議論されてきたが、通説は給付請求権が認められない理由として、国民の生存権保障のための具体的な方法が憲法では明らかでないため、立法府の判断に委ねられるとする見解 ^62^、生存権の内容が抽象的であり、内容を具現化する法律が必要であるとの見解が示されてきた ^63^。しかし、具体的請求権を認めないことは、生存権が憲法に規定されている意味に大きな疑問を生じさせる。また法学や司法は、憲法の視点から生存権を検討すべきであり、立法への過剰な委任は望ましくない ^64^。また、抽象的な規定を具体化するのが法学、司法の役割であると考えられ、憲法規定の抽象性を根拠として具体的請求権が否定されるべきではない ^65^。よって、生存権の内容を法学・司法が具体化し、憲法を基盤とした具体的請求権が認められるべきである。なお、従来では生活保護などの給付請求権が議論されてきたが、生存権保障の方策は給付に限らない。憲法 25 条 2 項は、「国は、すべての生活部面について、社会福祉、社会保障及び公衆衛生の向上及び増進に努めなければならない。」と規定している。よって、「公衆衛生の向上及び増進」のために必要な方策を請求することも、憲法 25 条に基づき可能なはずである。上記の請求がなされた際には、公衆衛生の保持を目的とした個人の自由の制限が正当化されうる。

第三に、憲法 13 条に規定されている「公共の福祉」は、個人の自由を制限する根拠となる。同条は、国民の権利が「公共の福祉に反しない限り、（中略）最大の尊重を必要とする」と規定しており、公共の福祉に適合しない場合、自由・人権が制限されうる。判例は公衆浴場法事件や薬局距離制限事件等を通して、公共の福祉概念に国民の保健・環境衛生が含まれるとしてきた ^66^。また学説でも、他者の権利・利益確保の文脈で、公共の福祉に公衆衛生が含まれると考えられており ^67^、これは妥当であろう。よって、公衆衛生の保全を目的とする COVID-19 対策は公共の福祉に含まれ、個人の自由の制限が正当化されうる。なお日本には、感染力が低いと認識されていたにもかかわらず、公共の福祉の名の下にハンセン病患者の隔離が行われた歴史があり ^68^、公共の福祉と感染症の関係性については慎重な検討が必要である。

## IV. COVID-19対策への適用

以上では、日本国憲法における個人の自由を保障する概念及び個人の自由を制限しうる概念を紹介した。本節ではそれぞれの概念を COVID-19 対策に適用し、個人の自由が保障されるべきか、もしくは COVID-19 対策のための自由の制限が認められるべきかを検討する。

まず、2021 年 1 月現在の罰則のない要請が憲法上認められるかを検討する。営業の自由及び移動の自由は、居住、移転及び職業選択の自由を規定する憲法 22 条、財産権を規定する 29 条、人格権を規定する 13 条が関連している。これらの人権は公共の福祉の制約を受けるが、権利の性質により制約の程度が異なると考えられている。判例、通説では、精神的自由についてはより厳格な基準で公共の福祉を捉え、経済的自由についてはより緩やかな基準で公共の福祉を捉える、「二重の基準論」が有力になっている ^69^。経済的自由が精神的自由と比較して社会的影響が大きいことに鑑みると、経済的自由に対してより強度な公共の福祉による制約を課すことは妥当である。営業の自由及び移動の自由の根拠となる居住、移転及び職業選択の自由、財産権は経済的自由に含まれると考えられるため ^70^、精神的自由権よりも緩やかな基準で公共の福祉を捉え、公共の福祉による一定程度の自由の制約が許容される。COVID-19 対策は公衆衛生の保全を目的としたものであり、公衆衛生の保全は公共の福祉に適合すると考えられるため、営業の自由及び移動の自由を経済的自由として捉えれば、その制限は正当化される。

なお、営業の自由及び移動の自由を人格権の一部として位置付ければ、公共の福祉がより厳格に捉えられ、COVID-19 対策よりもこれらの自由が優先されるであるとの議論もありうる。しかし、営業の自由及び移動の自由に人格権の側面が認められるとしても、精神的自由である人格権を基盤としてこれらの自由を認めることは困難である。第一に、営業の自由と「人格的価値」との関係性を認めた薬事法違憲判決では、営業の自由と人格的価値としての関連性に言及した後に、職業は「社会的相互関連性が大きい」ため、「公権力による規制の要請がつよ」いとしている ^71^。よって、営業の自由の主な規範的基盤は経済的自由権であり、営業の自由を COVID-19 対策より優先させることは困難である。第二に、移動の自由もその社会的影響を考慮すべきである。確かに従来、個人の移動の社会的な影響は限定的に捉えられてきた。しかし COVID-19 には無症状者が存在し ^72^、感染者が明らかでない。そのため、移動の制限が COVID-19 の感染拡大予防のために重要だと考えられる。このような社会的な影響があることに鑑みれば、COVID-19 対策としての移動の自由の制限は許容される。

その上で、生命権、生存権・公衆衛生の視点からすれば、COVID-19 による死のリスクを減らす方策を国家が採る必要がある。特に生命権は人権の基盤となるため、本質的にその保障が最も重要である。よって、自由が制限されたとしても公衆衛生の保全による生命権、生存権の保障が必要である。検討すべき点として、休業や営業時間短縮の要請・指示及び外出自粛要請が、COVID-19 対策に必要であるのか、という問題が挙げられる。筆者は感染症対策の専門家ではないため、信頼に足る自らの見解を示すことはできない。しかし、2020 年 12 月時点で新型コロナウイルス感染症対策分科会は、主な感染源として飲食の場が挙げられるとの見解を示しており ^73^、飲食店の休業や営業時間短縮の要請・指示には一定の意味があると考えられる。また、人が COVID-19 感染拡大の媒体となるため、移動の自粛要請にも一定の有効性があると考えられる ^74^。

最後に、罰則を伴う措置について検討する。日本国憲法は罰則のある外出制限、休業や営業時間短縮の措置を許容していると考えられる。COVID-19 対策が公共の福祉に適合すれば、罰則を禁止する根拠を見いだすことは困難だからである。とはいえ、憲法が罰則のある休業や営業時間短縮の要請・指示を求めているわけでもない。すなわち、罰則の有無は立法に委ねられている。しかし罰則を導入する際には、行政法における比例原則が適用される必要がある。比例原則の内容として、目的適合性の原則、必要性の原則、狭義の比例性の原則が挙げられる ^75^。よって、罰則は必要最小限で、かつ法益に与える影響が不釣り合いであってはならない。

## おわりに

本稿は、COVID-19 対策に関わる憲法上の論点を「個人の自由を保障する概念」及び「個人の自由を制限しうる概念」に整理し直した上で、COVID-19 対策が公共の福祉に適合すると捉えられ、特措法に基づく休業や営業時間短縮の要請・指示、外出自粛要請に憲法上の制約はないと論じた。その上で、生命権・生存権の議論を基盤として、国家による積極的な COVID-19 対策が求められると述べた。また、比例原則を考慮する必要はあるものの、憲法は罰則のある措置を許容しているとまとめた。よって、罰則のない特措法に基づく措置は、憲法による要請でなく、立法府の判断により規定されたものである。

最後に今後の検討課題として、2021 年 1 月以降の導入が検討されている罰則の意義と、ポスト・コロナ時代の国家観と憲法を挙げる。第一に、罰則導入の意義を検討する必要がある。罰則導入を検討する一義的な目的は、感染拡大防止のための休業もしくは営業時間の短縮の徹底であると考えられる ^76^。その上で、罰則の社会的な意義についても検討する必要がある。従来の特措法には罰則規定が存在しないが、2020 年 4 月の緊急事態宣言発出以降、営業自粛要請を遵守していないと思われる店舗を私人が攻撃する、「コロナ自警団」や「自粛警察」と呼ばれる現象が起きた ^77^。心理学者の榎本博明は、罰則を課した欧米では、自由の制限への不満の矛先が政府に向かったのに対し、日本では罰則のない自己犠牲を求める要請であったために、不満の矛先が要請を遵守しなかった市民に向かったと述べている ^78^。「コロナ自警団」の現象は、公的な罰則がなくとも社会的な制裁が課されうることを示している。社会的な制裁が横行することは、「正統な物理的暴力行使の独占」が期待・要求される近代国家において望ましくないであろう ^79^。罰則導入により感染拡大防止を徹底できるのか、また社会的制裁を減少させることができるのか、を今後分析する必要がある ^80^。

第二に、ポスト・コロナ時代の国家観と憲法について検討する必要がある。従来日本の憲法学は、大日本帝国憲法下の経験から自由の制限に抑制的な立場をとってきた ^81^。また特措法も、2012 年の成立以降罰則規定が不在であった。これらの前提には、国家の権限強化への警戒感がある ^82^。しかし COVID-19 は、この国家観に変化をもたらす可能性がある。生命権保障のためには国家による積極的な COVID-19 対策が求められる。また、国民が国家の権限強化を求める動きもある。2020 年 6 月に NHK が実施した世論調査では、62% が外出禁止や休業を強制できる法改正が必要であると回答している ^83^。また 2021 年 1 月に NHK が実施した世論調査では、48% が特措法への罰則の明記に賛成と回答しており ^84^、罰則のある措置に肯定的な意見が一定数あることを示している ^85^。世論が実際にどの程度罰則のある措置を望んでいるのかは明らかでない。国家に強力な権限を認めることには全体主義につながるとの懸念があり ^86^、国民が国家による罰則を望む現象が、近代立憲主義の視点から危険視されるのは当然である。しかし未知のウイルスに恐怖を感じ、その克服のために強力な権限を求めるのは、社会の自然な反応ということもできよう。全体主義の歴史に学び、政治による恣意的な権力の集中を避けつつ、科学者の専門知を基盤として、国家が生命権保障のために必要な措置を検討する必要がある。感染症対策における国家の役割、憲法の役割については、今後さらなる議論が求められる。また、COVID-19 は憲法が直面する新たな問題である点も重要である。従来想定されてきた「緊急事態」における「敵」は、人間により構成される国家であり、「敵」の認定には政治的判断が不可避である。その一方で、COVID-19 等感染症対策における「敵」はウイルスであり、科学的な専門知により根拠づけられる ^87^。憲法（学）が必ずしも想定してこなかった新たな、かつ人間社会に与える影響が巨大な「敵」が出現する中で、憲法学が描く国家観を今後再検討する必要がある。

COVID-19 は、大規模かつ長期化する感染症と向き合うことを迫っており、日本国憲法にとって新たな課題である。新型コロナウイルスを含む新興の人獣共通感染症は増加傾向にあるとの指摘もあり ^88^、今後も日本社会は感染症と向き合う必要がある ^89^。憲法の枠組みに基づき法的な検討を行うとともに、日本社会の特性も鑑みて、有効な感染症対策により生命権を保障する、新たな国家観を構想していく必要がある。

## Footnotes

^1^
See, Hannah Ritchie, Esteban Ortiz-Ospina, Diana Beltekian, Edouard Mathieu, Joe Hasell, Bobbie Macdonald, Charlie Giattino, and Max Roser, “Policy Responses to the Coronavirus Pandemic,” OurWorldInData.org, accessed on 2 January 2021, https://ourworldindata.org/policy-responses-covid.

^2^
以下の江藤論文以外に、日本国憲法における「公共の福祉」の概念により、強制力のある COVID-19 対策が可能であるとの議論はあるが （井上達夫「コロナ・ラプソディー― パンデミックが暴く『無責任の体系』」法と哲学 6 号（2020 年） 36-37 頁。Lawrence Repeta, “The Coronavirus and Japan’s Constitution: Article 41 Provides the Government with Sufficient Power to Take Aggressive Action,” *The Japan Times*, 14 April 2020, https://www.japantimes.co.jp/opinion/2020/04/14/commentary/japan-commentary/coronavirus-japans-constitution/ (accessed on 10 January 2021).）、憲法上の関連する概念を包括的に検討したものでない。

^3^
江藤祥平「匿名の権力――感染症と憲法」法律時報 92 巻 9 号 （2020 年） 70-77 頁。

^4^
同論文 70 頁。

^5^
菅首相は 2021 年 1 月 4 日の記者会見で、罰則を盛り込んだ特措法改正案を 2021 年 1 月に開会する通常国会に提出すると述べた。首相官邸「令和 3 年 1 月 4 日　菅内閣総理大臣記者会見」 https://www.kantei.go.jp/jp/99_suga/statement/2021/0104kaiken.html （2021 年 1 月 4 日、2021 年 1 月 4 日アクセス） 。

^6^
なお、本稿が対象とするのは 2021 年 1 月現在の特措法に基づく罰則のない措置であるが、罰則が導入されても、特措法の構造が変わらない限り主な COVID-19 対策と憲法をめぐる論点は変わらないと考えられる。

^7^
感染症法に関する議論は、太田匡彦「新型コロナウイルス感染症にテストされる感染症法 （上） 」法律時報 92 巻 9 号 （2020 年） 85-91 頁、太田匡彦「新型コロナウイルス感染症にテストされる感染症法 （下） 」法律時報 92 巻 11 号 （2020 年） 84-90 頁を参照。

^8^
新型インフルエンザ等対策研究会『逐条解説　新型インフルエンザ等対策特別措置法』 （中央法規出版、2013 年） 3-6 頁。なお本書籍は、COVID-19 の感染拡大を受け、中央法規出版ウェブサイト内において期間限定で無料公開されている。無料公開してくださった中央法規出版に感謝申し上げる。

^9^
新型インフルエンザとは、新たに人から人に伝染する能力を有することとなったウイルスを病原体とするインフルエンザを指す。再興型インフルエンザとは、かつて世界規模で流行したが、その後長期間流行しなかったインフルエンザで、再興したものを指す。新感染症とは、人から人に伝染する未知の疾病のうち、以下の三要件を満たすものをいう。第一に、当該疾病にかかった際の病状の程度が重篤であるもの、第二に、当該疾病のまん延が国民の生命、健康に重大な影響を与える恐れがあるもの、第三に、全国的かつ急速なまん延の恐れがあるもの、である。特措法 2 条 1 号、感染症法 6 条 7 項、同条 9 項参照。なお、感染症法は既知の感染症も網羅している点が、特措法との違いである。

^10^
特措法 1 条。

^11^
一般社団法人アジア・パシフィック・イニシアティブ『新型コロナ対応民間臨時調査会調査・検証報告書』 （ディスカヴァー・トゥエンティワン、2020 年） 133 頁。

^12^
同書 133-134 頁。

^13^
同書 134 頁。感染症法 19 条、20 条、26 条。

^14^
同書 134 頁。検疫法 13 条 1 項。

^15^
首相官邸「令和 2 年 2 月 27 日　新型コロナウイルス感染症対策本部 （第 15 回） 」 https://www.kantei.go.jp/jp/98_abe/actions/202002/27corona.html （2020 年 2 月 27 日、2021 年 1 月 2 日アクセス） 。

^16^
当時の経緯については、一般社団法人アジア・パシフィック・イニシアティブ・前掲注 11 、134-137 頁も参照。

^17^
新型インフルエンザ等対策研究会・前掲注 8 、8 頁。

^18^
特措法 15 条、22 条。

^19^
特措法 32 条。

^20^
新型インフルエンザ等対策研究会・前掲注 8 、117-118 頁。

^21^
特措法 45 条 1 ・ 2 項。この「要請」には不利益処分がなく、行政手続法 2 条 1 項 6 号に規定される「行政指導」と考えられる。内閣官房新型コロナウイルス感染症対策推進室長「事務連絡　第 45 条の規定に基づく要請、指示及び公表について」 （令和 2 年 4 月 23 日） https://corona.go.jp/news/pdf/yousei_shiji_0423.pdf （2021 年 1 月10 日アクセス） 。よって、法的義務は課されないと考えられている。参照、髙木光・常岡孝好・須田守『条解行政手続法』 （弘文堂、2017 年） 30 頁。また緊急事態措置の対象となった都道府県知事は、臨時の医療施設の開設のために私有地を使用することができる。特措法 48 ・ 49 条。

^22^
特措法 45 条 3 項。参照、内閣官房新型コロナウイルス感染症対策推進室長・前掲注 21 。

^23^
特措法 45 条 4 項。

^24^
片山は、政府対策本部が設置され、緊急事態宣言が発出されていない際に適用される特措法 24 条は、医師会や看護協会、大学の感染症研究者の協力を得るような事態を想定しており、営業停止などを要請する根拠とはならないとしている。片山善博「連載 128 　片山善博の『日本を診る』　『緊急事態宣言』をめぐる法律のとんでもない読み間違い」世界 2020 年 7 月 （934） 号 （2020 年） 105-106 頁。片山の主張するように、政府対策本部が設置され緊急事態宣言が発出されていない時は「身構える段階」であり、緊急事態宣言発出時とは性質が異なる。しかし、同法 24 条に基づく要請は公表の規定がない一方で、緊急事態宣言発出時に適用される同法 45 条に基づく要請・指示は公表を義務付けている。重要なのは、片山も指摘するように、45 条に基づく要請・指示を遵守しない場合に公表されるのではなく、要請・指示が行われた時点で公表される点である。同法 24 条に基づく休業や営業時間短縮の要請が行わなければ、45 条に基づいて休業や営業短縮が行われた場合、突然公表されることになり、不合理である。そのため、24 条によっても休業や営業時間短縮の要請が可能であり、緊急事態宣言により、公表が義務付けられると考える方が自然であろう。

^25^
都道府県知事が政府の基本的対処方針に基づく「総合調整」 （特措法 20 条 1 項） に従わない場合、政府は「必要な指示」 （同法 33 条 1 項） を出すことができる。なお、安田は緊急事態宣言発出時に着目して、基本的対処方針の役割に言及しているが（安田理恵「日本の新型コロナウイルス感染症対策からみた国、都道府県および住民の関係」法学セミナー788 号 （2020 年） 6-7 頁。）、政府対策本部が設置されている段階であれば、緊急事態宣言が発出されずとも基本的対処方針は同様の役割を果たすと考えられる。

^26^
特措法 5 条。

^27^
2020 年 1 月 30 日に閣議決定により新型コロナウイルス感染症対策本部が設置されたが （「新型コロナウイルス感染症対策本部の設置について」 （令和 2 年 1 月 30 日閣議決定） https://www.cas.go.jp/jp/influenza/konkyo_corona.pdf （2021 年 1 月 3 日アクセス） 。） 、同年 3 月 14 日に、特措法に基づく政府対策本部となった。

^28^
nippon.com 「新型コロナウイルス感染症流行・ 3 月の主な動き」 https://www.nippon.com/ja/japan-data/h00730/ （2020 年 5 月 21 日、2021 年 1 月 4 日アクセス） 。

^29^
一般社団法人アジア・パシフィック・イニシアティブ・前掲注 11 、141 頁。

^30^
4 月 7 日に東京、神奈川、埼玉、千葉、大阪、兵庫、福岡の 7 都府県が緊急事態措置の対象となり、4 月 16 日にその対象が全国に拡大した。 5 月 14 日に北海道、東京、神奈川、埼玉、千葉、大阪、京都、兵庫を除く 39 県が緊急事態措置の対象から除外された。 5 月 21 日に大阪、京都、兵庫が対象から除外され、5 月 25 日に北海道、東京、神奈川、埼玉、千葉も除外され、緊急事態宣言が解除された。同書、457-458 頁。

^31^
内閣官房新型コロナウイルス感染症対策推進室「新型コロナウイルス感染症 緊急事態宣言の実施状況に関する報告」 （令和 2 年 6 月） 3 頁、 https://corona.go.jp/news/pdf/kinkyujitaisengen_houkoku0604.pdf （2021 年 1 月 3 日アクセス） 。

^32^
同報告書、3 頁。

^33^

例えば東京都は、緊急事態宣言解除後の 2020 年 7 月に不要不急の外出自粛要請を行った。 nippon.com 「新型コロナウイルス感染症流行・ 7 月の主な動き」 https://www.nippon.com/ja/japan-data/h00870/ （2020 年 11 月 19 日、2021 年 1 月 4 日アクセス） 。

^34^
例えば東京都は緊急事態宣言解除後も休業要請を継続し、段階的に要請を緩和することとした。東京都「新型コロナウイルス感染症を乗り越えるためのロードマップ〜「新しい日常」が定着した社会の構築に向けて〜」 https://www.metro.tokyo.lg.jp/tosei/hodohappyo/press/2020/05/22/documents/11_00_1.pdf （2021 年 1 月 4 日アクセス） 。また大阪府は 2020 年 8 月に、府の指定した対策を講じない接待を伴う飲食店やカラオケ店に休業を要請し、それ以外の飲食店にも営業時間短縮を要請した。 nippon.com ・前掲注 33 。

^35^
首相官邸・前掲注 5 。

^36^
内閣官房新型コロナウイルス感染症対策推進室「新型コロナウイルス感染症緊急事態宣言」 https://corona.go.jp/emergency/ （2021 年 1 月 10 日アクセス） 。

^37^
同ウェブサイト。なお、新型コロナウイルス感染症対策本部は 2021 年 1 月現在も設置されており、緊急事態措置の対象となっていない都道府県知事は特措法 24 条に依拠した要請が可能である。

^38^
近代立憲主義については、長谷部恭男『憲法講話―― 24 の入門講義』 （有斐閣、2020 年） 1-14 頁を参照。

^39^

最高裁判所大法廷判決昭和 47 年 11 月 22 日刑集 26 巻 9 号 586 頁。芦部信喜 （高橋和之補訂） 『憲法〔第 7 版〕』 （岩波書店、2019 年） 233 頁。佐藤幸治『日本国憲法論〔第 2 版〕』 （成文堂、2020 年） 335 頁。長谷部恭男『憲法〔第 7 版〕』 （新世社、2018 年） 241 頁。

^40^
浦部法穂『憲法学教室〔第 3 版〕』 （日本評論社、2016 年） 238-239 頁。渋谷秀樹『憲法〔第 3 版〕』 （有斐閣、2019 年） 295 頁。なお後述の通り、職業選択の自由及び財産権はともに経済的社会権であり、営業の自由の根拠が 22 条のみであっても、22 条及び 29 条であっても、本質的な違いはあまりないと思われる。よって本稿では、採るべき説についての議論は行わない。同様の見解は野中俊彦・中村睦男・高橋和之・高見勝利『憲法I〔第 5 版〕』 （有斐閣、2012 年） 472 頁を参照。

^41^
最高裁判所大法廷判決昭和 50 年 4 月 30 日民集 29 巻 4 号 572 頁。

^42^
佐藤・前掲注 39 、335 頁。江藤・前掲注 3 、74 頁。

^43^

憲法 13 条と人格権の関連については、渋谷・前掲注 40 、183 頁、野中・中村・高橋・高見・前掲注 40 、269-273 頁を参照。

^44^
浦部・前掲注 40 、243 頁。野中・中村・高橋・高見・前掲注 40 、459 頁。なお渋谷は、憲法 22 条 2 項に規定される「外国に移住する自由」に、外国に一時旅行する自由が含まれるべきであるとの判例に言及しつつ、居住・移転の自由を規定する憲法 22 条 1 項が移動の自由を保障すると述べている。渋谷・前掲注 40 、224-225 頁。

^45^
浦部・前掲注 40 、243 頁。野中・中村・高橋・高見・前掲注 40 、458 頁。

^46^
櫻田誉「憲法における生命権の保障-救命救急医療との関連を中心として-」関西大学法学論集 37 巻 2 ・ 3 号 （1987 年） 4 頁。

^47^
石村修「基本権の体系における生きる権利の意味」法学新報 96 巻 11 ・ 12 号 （1990 年） 98-107 頁。

^48^
Noel Williams, *The Right to Life in Japan* (London and New York: Routledge, 1997).

^49^
小林直樹「人権価値を根底から考える――哲学的人間学の視点から」憲法問題 9 号 （1998 年） 156-159 頁。

^50^
山内敏弘「基本的人権としての生命権の再構成」杉原泰雄先生古稀記念論文集刊行会編『二一世紀の立憲主義-現代憲法の歴史と課題-』 （頸草書房、2000 年） 325-345 頁。加筆を経て、山内敏弘『人権・主権・平和――生命権からの憲法的省察――』 （日本評論社、2003 年） 2-32 頁にも収録されている。生命権論のその後の展開については、山内敏弘「基本的人権としての生命権の再定位」龍谷法学 53 巻 2 号 （2020 年） 43 頁を参照。

^51^

芦部・前掲注 39 、120 頁。佐藤・前掲注 39 、196 頁。野中・中村・高橋・高見・前掲注 40 、272 頁。浦部・前掲注 40 、45 頁。

^52^
判例では、憲法 13 条に基礎付けられる権利・自由として、私生活上の自由 （最高裁判所大法廷判決昭和 44 年 12 月 24 日刑集 23 巻 12 号1625 頁） と人格権 （最高裁判所大法廷判決昭和 61 年 6 月民集 40 巻 4 号 872 頁） が指摘されている。渋谷・前掲注 40 、183-184 頁。

^53^
山内・前掲注 50 （「基本的人権としての生命権の再定位」） 、1 頁。なお、詳細には論じられていないものの、辻村や渋谷が生命権の議論に触れており、生命権について議論されつつある。辻村みよ子『憲法〔第 6 版〕』 （日本評論社、2018 年） 140 頁。渋谷・前掲注 40 、185-187 頁。

^54^
山内・前掲注 50 （『人権・主権・平和-生命権からの憲法的省察』） 、4 頁。

^55^
同書、4 頁。

^56^
例えば、生田勝義「死刑と生命権 （再論） ――死刑憲法 13 条違憲論は死刑廃止に役立つか――」立命館法学 365 号 （2016 年） 110-141 頁は死刑の観点から生命権を検討している。

^57^
国家による保護義務を認めることは、国家の不当な介入を招く恐れが大きいとの批判もある。芦部は国家による介入の限定が困難であるとして、日本国憲法の解釈に国家の保護義務を導入することへの懸念を示している。芦部信喜『宗教・人権・憲法学』 （有斐閣、1999 年） 230 頁。しかし、生命の保護が人権保障の基盤であることを考えると、生命保護のための国家による積極的行動は必要であろう。また山内は、近代憲法の思想的基盤であるロックの社会契約論から、国家の市民の保護義務を導き出すことができると述べる。山内・前掲注 50 （「基本的人権としての生命権の再定位」） 、25-26 頁。なお、生命権の議論の先駆者と言える櫻田誉は、日本における救命救急医療体制が不十分であるとの認識から生命権の議論を行っており、COVID-19 対策に関する生命権の議論にも示唆的である。櫻田・前掲注 46 、254-273 頁。

^58^
厚生労働省「新型コロナウイルス について オープンデータ 死亡者数」 https://www.mhlw.go.jp/content/death_total.csv （2021 年 1 月 11 日アクセス） 。

^59^
根岸も、日本政府が生命を保護する義務を十分に果たしていないと評価している。 Yota Negishi, “Japan,” Bonavero Institute of Human Rights, *Bonavero Report 7/2020: A Human Rights and Rule of Law Assessment of Legislative and Regulatory Responses to the COVID-19 Pandemic across 27 Jurisdictions*, 277. ただ、例えば日本における癌による死者は 2018 年に 373,584 人であり （国立がん研究センター 癌情報サービス「最新がん統計」 https://ganjoho.jp/reg_stat/statistics/stat/summary.html （2020 年 7 月 6 日、2021 年 1 月 5 日アクセス。）） 、COVID-19 が他の要因と比較して圧倒的な数の死者の要因となっているわけではないことには留意が必要である。何が生命権の侵害となるかとの見解には、社会における価値判断が必要であろう。本稿は、COVID-19 が感染症であり、一人の感染が他者の生命にも影響を及ぼす可能性がある点、COVID-19 対応のために医療体制が逼迫している点を踏まえ （NHK 「コロナ重症者治療の医師『病床はほぼ満床 年末年始は静かに』」 https://www3.nhk.or.jp/news/special/coronavirus/medical/detail/detail_71.html （2020 年 12 月 28 日、2021 年 1 月 4 日アクセス） 。） 、COVID-19 が生命権への脅威であると論じる。

^60^
NHK・前掲注 59 。

^61^
通説は、芦部・前掲注 39 、120 頁、浦部・前掲注 40 、250-251 頁を参照。判例は、憲法 25 条が「すべての国民が健康で文化的な最低限度の生活を営み得るように国政を運営すべきことを国の責務として宣言したにとどまり、直接個々の国民に対して具体的権利を賦与したものではない」とする。最高裁判所大法廷判決昭和 42 年 5 月 24 日民集 21 巻 5 号 1043 頁。

^62^
浦部・前掲注 40 、251 頁。

^63^
芦部・前掲注 39 、279 頁。

^64^
司法が立法の視点を考慮に入れることの問題点については、渋谷・前掲注 40 、278 頁を参照。

^65^
例えばいわゆる「一票の格差」の問題について最高裁は、投票価値の平等が「他の政策的な目的ないし理由との関連において調和的に実現されるべきもの」とし （最高裁判所大法廷判決 昭和 51 年 4 月 14 日民集 30 巻 3 号 223 頁） 、立法府の役割を認めつつも違憲審査を行ってきた。これまでの主な判例については芦部・前掲注 39 、146-153 頁を参照。また学説でも、投票価値が二対一以上になることは法の下の平等の観点から違憲であるとの見解が示されている。芦部・前掲注 39 、145 頁。生存権も同様に、法学・司法による基準の具体化が望ましいと考えられる。

^66^

渋谷秀樹「『公共の福祉』とは何か」岡田信弘・笹田栄司・長谷部恭男編『憲法の基底と憲法論――思想・制度・運用――』 （信山社、2015 年） 45 頁。

^67^
同書、55-56 頁。

^68^
参照、財団法人日弁連法務研究財団 ハンセン病問題に関する検証会議『ハンセン病問題に関する検証会議 最終報告書』 （2005 年） 129 頁、 https://www.mhlw.go.jp/topics/bukyoku/kenkou/hansen/kanren/4a.html （2021 年 1 月 10 日アクセス） 。

^69^
芦部・前掲注 39 、104-107 頁。最高裁判所大法廷判決昭和 50 年 4 月 30 日民集 29 巻 4 号 572 頁。

^70^
芦部・前掲注 39 、233-250 頁。

^71^
最高裁判所大法廷判決昭和 50 年 4 月 30 日民集 29 巻 4 号 572 頁。

^72^

Yahoo! Japan ニュース「『感染させる人の約半数は無症状。若い人はリアリティを知ってほしい』　尾身会長の緊急メッセージ」 https://news.yahoo.co.jp/articles/0a1ea2579b9116057fec9db92fe50f88638ca603 （2020 年 12 月 31 日、2021 年 1 月 4 日アクセス） 。

^73^
首相官邸「令和 2 年 12 月 25 日 新型コロナウイルス感染症に関する菅内閣総理大臣記者会見」 https://www.kantei.go.jp/jp/99_suga/statement/2020/1225kaiken.html （2020 年 12 月 25 日、2021 年 1 月 4 日アクセス） 。同分科会は、新型インフルエンザ等対策閣僚会議内の、新型インフルエンザ等対策有識者会議に設置されている。内閣官房「新型インフルエンザ等対策有識者会議の開催について」 （令和 2 年 7 月 3 日一部改正） https://www.cas.go.jp/jp/seisaku/ful/yusikisyakaigi/konkyo.pdf （2021 年 1 月 4 日アクセス） 。

^74^
本稿では詳細に立ち入らないが、科学的に望ましい対処法が明らかでない場合の対策も重要である。変異した新型コロナウイルスも確認されており、ウイルスの特徴が変化する可能性もあるからである。こうした場合には、国際的に環境問題等に用いられる「予防原則 （precautionary principle） 」を導入することも、生命権を保障するために検討する必要があろう。予防原則とは、「科学的知見が十分に備わっていなくても、ひとたび問題が生じたとき、深刻かつ不可逆的な被害が発生する場合に、何もしないのではなく、必要な対策をとるべきである、とする法原則」である。松本和彦「公法解釈における諸原理・原則の対抗――憲法学から見た比例原則・予防原則・平等原則」公法研究 81 号 （2019 年） 67 頁。予防原則については批判もあるが（参照、西原博史「リスク社会・予防原則・比例原則」ジュリスト 1356 号 （2008 年） 75 頁。） 、生命が人権の基盤であり、一度失われると回復不可能であることを考えると、予防原則を採る必要性がある。感染リスクがないことが立証されるまで、感染リスクとなりうる行為の積極的な抑制を検討する必要があろう。なお、予防原則は後述の比例原則と緊張関係を孕む可能性があり、過剰な自由の制限、罰則を回避しつつ、生命のリスクをいかに軽減するかは今後の検討課題である。

^75^
高橋明男「比例原則審査の可能性」法律時報 85 巻 2 号 （2013 年） 21 頁。

^76^
神奈川県知事は 2020 年 12 月に、営業時間短縮の要請に 2 割しか応じていないとの県職員の感触を紹介している。東京新聞「時短営業『 2 割しか応じていない』と危機感　神奈川県・黒岩知事、根拠は『県職員が街を見回った実感』」 https://www.tokyo-np.co.jp/article/75931 （2020年 12 月 22 日、2021 年 1 月 10 日アクセス） 。なお外出制限に関しては、2021 年 1 月 10 日現在、罰則導入は検討されていないようである。

^77^
参照、鴻上尚史・佐藤直樹『同調圧力:日本社会はなぜ息苦しいのか』 （講談社、2020 年） 128-132 頁。

^78^
週刊現代「『空気を読む日本人』ほど、なぜか『自粛警察』になってしまう“意外なワケ”」 https://gendai.ismedia.jp/articles/-/78675?page=4 （2020 年 12 月29 日、2021 年 1 月 10 日アクセス） 。日本における同調圧力と「コロナ自警団」については、鴻上・佐藤・前掲注 77 、130 頁参照。

^79^
Max Weber, “Politics as a Vocation,” in *The Vocation of Lectures “Science as a Vocation” “Politics as a Vocation*, *”* ed. David S. Owen and Tracy B. Strong, trans. Rodney Livingstone (Indianapolis and Cambridge: Hackett Publishing Company, 2004), 33. 原文では本箇所に強調が加えられている。日本語訳は、マックス・ヴェーバー『職業としての政治〔改版〕』 （脇圭平訳、岩波書店、2020 年） 8-9 頁を参考にした。

^80^
2020 年 5 月の時点で江島は、自粛要請による対策が、政府に強大な権限を与えずとも感染拡大を予防できるか否かを占う試金石になると述べている。 Akiko Ejima, “Japan’s Soft State of Emergency: Social Pressure Instead of Legal Penalty,” in *Verfassungsblog on Matters Constitutional*, 13 May 2020, https://verfassungsblog.de/japans-soft-state-of-emergency-social-pressure-instead-of-legal-penalty/ (accessed on 10 January 2021). 2021 年 1 月現在では、「コロナ自警団」の事例、休業もしくは営業時間短縮の要請に応じる事業者が不十分である事例から、自粛要請による対策の効果は慎重に判断すべきだろう。

^81^
大日本帝国憲法では国家緊急権が規定されていた一方で、日本国憲法には同様の規定がないこともその例であろう。参照、高橋和之『立憲主義と日本国憲法〔第 5 版〕』 （有斐閣、2020 年） 、470-471 頁。

^82^
注 57 も参照。

^83^
NHK「外出禁止や休業を強制できる法改正必要 62% NHK 世論調査」 https://www3.nhk.or.jp/news/html/20200623/k10012480041000.html （2020 年 6 月 23 日、2021 年 1 月 1 日アクセス） 。また 2020 年 5 月に JNN が実施した世論調査では、56% が罰則規定の導入に賛成している。 TBS NEWS JNN 世論調査「特措法をめぐる法改正について?」 https://news.tbs.co.jp/newsi_sp/yoron/backnumber/20200509/q2-7.html （2020 年 5 月 9-10 日、2021 年 1 月 10 日アクセス） 。

^84^
NHK「2021 年 1 月 政治意識月例電話調査」 https://www.nhk.or.jp/senkyo/shijiritsu/pdf/aggregate/2021/y202101.pdf （2021 年 1 月 13 日アクセス） 。

^85^
なお、2020 年 11-12 月に朝日新聞が実施した世論調査では、飲食店の休業指示に従わなかった場合の罰金に賛成したのは 33% に留まる。朝日新聞「休業 従わない飲食店に… 『罰金』 33% 『判断尊重』 62% 　本社世論調査」 2021 年 1 月 10 日朝刊 3 面。しかし計 3 回の NHK や JNN による世論調査において半数以上もしくは半数近くが罰則に肯定的な意見を表明しているため、本稿では罰則に肯定的な意見が一定数あると評価した。

^86^
参照、江藤・前掲注 3 、77 頁。

^87^
しかし、科学的な知見にも政治的な要素が入り得ることには注意が必要である。

^88^
勝間靖「新型コロナウイルス感染症 （COVID-19） の感染予防と蔓延防止のための政策 ―ベトナム、タイ、ベルギーでの事例を中心として―」WASEDA ONLINE https://yab.yomiuri.co.jp/adv/wol/opinion/20200430.php （2020 年 4 月 30 日、2021 年 1 月 10 日アクセス） 。

^89^
その要因として、人間による世界各国での森林開発のため、野生動物のウイルスが人間社会に持ち込まれていることが指摘されている。湯本貴和「コロナ危機は生態系からの警告である」世界 2020 年 8 月 （935） 号 （2020 年） 111-112 頁。

## データ利用可能性

本論文は法学の論文であり、「生データ (raw data) 」は存在しない。書籍や論文として公開されている学説、判例等に基づいている。

