# COVID-19対策と日本国憲法が保障する人権：新型インフルエンザ等対策特別措置法に着目して

**DOI:** 10.12688/f1000research.50861.2

**Published:** 2021-09-14

**Authors:** Hajime Akiyama

**Affiliations:** 1 Faculty of Humanities and Social Sciences, University of Tsukuba, Tsukuba, Ibaraki, 305-8571, Japan

**Keywords:** 新型コロナウイルス感染症, 日本国憲法, 新型インフルエンザ等対策特別措置法, 人権, 営業の自由, 移動の自由, 生命権, 生存権, 公衆衛生, 公共の福祉, COVID-19, the Constitution of Japan, the Act on Special Measures for Pandemic Influenza and New Infectious Diseases Preparedness and Response, human rights, freedom of establishment, freedom of movement, right to life, welfare rights, public health, public welfare

## Abstract

2020年3月以来、日本においては新型インフルエンザ等対策特別措置法がCOVID-19対策の中心的な役割を果たしてきた。同法に基づき、休業や営業時間短縮の要請・指示・命令、外出自粛要請が行われてきた。これらには日本国憲法が保障する営業の自由や移動の自由を制限する側面がある。そこで本稿は、「COVID-19対策としての休業や営業時間短縮の要請・指示・命令及び外出自粛要請に、憲法上の制約もしくは要請はあるか」をリサーチ・クエスチョンとして検討を行った。また、2021年2月に罰則が導入されたことを踏まえ「罰則のある措置は憲法上認められるか」との論点も補完的に扱った。本稿は、日本国憲法が保障する人権を「個人の自由を保障する概念」及び「個人の自由を制限しうる概念」に分けて検討した。個人の自由を保障する概念としては、営業の自由と移動の自由が挙げられる。これらの自由は主に居住、移転及び職業選択の自由（憲法22条）及び財産権（同29条）により保障される。また、生命権（同13条）、生存権・公衆衛生（同25条）及び公共の福祉（同13条）への脅威となる個人の自由は制限されうる。本稿は、生命権、生存権・公衆衛生及び公共の福祉の観点から、日本国憲法は営業の自由及び移動の自由の制限するCOVID-19対策を許容しており、政府がCOVID-19に起因する生命へのリスクを低減させる責任を負っていると主張した。さらに比例原則を検討する必要はあるものの、憲法は罰則のある措置を許容していると論じた。

## Abstract (en)

Since March 2020, the Act on Special Measures for Pandemic Influenza and New Infectious Diseases Preparedness and Response has been a significant statute in dealing with COVID-19 in Japan. The Act mandates requests, instructions and orders for business suspension and shortened business hours, as well as stay-at-home requests. These measures limit freedom of movement and establishment, guaranteed rights under the Japanese Constitution. This article poses the following research question: “Does the Japanese Constitution allow measures against COVID-19 such as requests, instructions and orders for business suspension and shortened business hours, and stay-at-home requests?” It also asks: “Are measures with penalties allowed by the Constitution?” given the fact that the penalties were introduced in February 2021. This paper introduces constitutional concepts that guarantee or limit individual freedom. Concepts that guarantee individual freedoms include freedom of establishment and movement. These freedoms derive from the constitutional values of freedom to choose one’s occupation and choose and change one’s residence (Art. 22) and the right to own or hold property (Art. 29). Concepts that limit individual freedom include the right to life (Art. 13), welfare rights and public health (Art. 25), and public welfare (Art. 13). Individual freedom that threatens right to life, welfare rights and public health, and public welfare may not be guaranteed. This paper argues that the Constitution allows the measures against COVID-19 limiting freedom of establishment and movement from the perspectives of the right to life, welfare rights, public health, and public welfare, and the government is responsible for reducing the risk to life from COVID-19. It also argues that the Constitution permits measures with penalties, while proportionality needs to be considered.

## はじめに

2020 年から 2021 年にかけて新型コロナウイルス感染症 （以下、COVID-19） の感染確認者数が増加し、社会は大きな影響を受けている。COVID-19には無症状者が多く ^1^、感染者が明らかでないため、感染拡大予防のために外出を制限する国が多い。各国の COVID-19 対策を分析する Our World in Data によれば、2021 年 8 月 11 日現在、116 の国や地域で外出自粛要請や外出制限などの措置が取られている ^2^。個人の自由を制限するCOVID-19対策は、社会が感染症といかに向き合うべきかとの論点を想起させる。人権は今日、グローバルな規範として認識され、日本国憲法にも多くの人権規定が置かれている。そして歴史的・思想的基盤となったのが、個人の国家からの自由を保障する自由権概念であった。COVID-19対策は、従来個人の自由と考えられてきた営業や移動の自由を制限する側面があり、今日の社会における根本的な価値に疑問を投げかけている。

日本においても、COVID-19対策として営業や移動の自由には制限が課されてきた。2020 年 3 月以来「新型インフルエンザ等対策特別措置法 （以下、特措法） 」が COVID-19 対策の中心的な役割を果たしてきた。2021 年 2 月までは同法に基づき、休業や営業時間短縮の要請・指示、外出自粛要請が行われてきた。2021 年 2 月に同法が改正され、休業や営業時間短縮の命令が可能になり、罰則規定も設けられた。これらの要請・指示・命令には日本国憲法が保障する営業の自由や移動の自由を制限する側面がある一方で、COVID-19 対策が憲法に規定される公共の福祉に適合しており、自由の制限はやむを得ないとの見解もあり得る。

日本の COVID-19 対策の憲法上の論点については多様な論点が指摘されてきたが、COVID-19対策と憲法が保障する人権を包括的に議論した論文は、本論文の第 1 版 (version 1) 脱稿時点（2021 年 1 月 10 日）では限られていた ^3^。その中で、特に包括的な分析を行った論文として、2020 年に執筆された江藤祥平による論文が挙げられる ^4^。江藤は「公共の福祉」と「個人の人権」という図式を提示し、感染症対策のための私権の制限の合憲性について詳細かつ批判的に検討している。同論文は憲法における公衆衛生、表現の自由、営業の自由、個人の尊厳、自己決定権にとどまらず、国際人権法における健康への権利にも言及しており、多角的に COVID-19 対策について検討を行っている。しかし、江藤論文における分析に加えて、以下の二点の検討が必要である。第一に、憲法 13 条に規定される生命権を検討する必要がある。通説では生命権が十分に議論されてこなかったが、COVID-19 の感染拡大は人権の基盤である生命への脅威と捉えることができ、生命権の概念を含めて COVID-19 対策を検討し直す必要がある ^5^。第二に、「公共の福祉」と「個人の人権」という分類の妥当性を再検討する必要がある。江藤の述べる通り、この二項対立は憲法学では「お馴染みの図式」である ^6^。従来、人権保障が憲法の一義的な価値の一つであり、公共の福祉により人権が制限されうるとの説明がなされてきた ^7^。しかし COVID-19 対策においては、公共の福祉と人権でなく、人権規範同士が衝突することも想定される。例えば COVID-19 が生命権への脅威であると捉えれば、感染拡大予防策により江藤が「個人の人権」と呼ぶ自由権的な権利が制限される一方で、生命権が保障される可能性がある。換言すれば、従来「公共の福祉」として捉えられてきた自由権の制限により保障される権利もまたあるのである ^8^。すなわち人権には国家による干渉に否定的な自由権の側面と、国家による積極的な行動を求める社会権の側面があるため、人権における自由権の価値を相対化する必要がある。人権の制約規範であると考えられてきた公共の福祉の内容を、憲法の一義的な価値である人権として位置付け直すことは、自由の制限への憲法上の意味を評価するために重要である。よって人権を的確に理解するために、「個人の自由を保障する概念」及び「個人の自由を制限しうる概念」に整理し直し、COVID-19 対策の憲法上の論点を再検討する必要がある。

本稿のリサーチ・クエスチョンは、「COVID-19 対策としての休業や営業時間短縮の要請・指示・命令及び外出自粛要請に、憲法上の制約もしくは要請はあるか」である。また、2021 年 2 月に特措法が改正され、罰則規定が導入されたことを踏まえ 、「罰則のある措置は憲法上認められるか」との論点も検討する。

なお、本稿は特措法に基づく対応に着目して検討する。特措法以外にも、感染症法上の指定感染症 ^9^、検疫法上の措置、予防接種法上のワクチン等の論点があるが、これらは別稿に譲りたい。その上で、本稿は特措法に基づく対策の総論的な論点の整理に留め、各論的な議論の詳細には立ち入らない。例えば首長による要請・指示・命令については、行政法の視点を導入する必要があるが、論点が散逸するため詳細には扱わない。また、本稿は 2021 年 1 月 10 日以前の情報をもとに執筆した第1版を基盤としつつ、第1版への査読コメントを踏まえて、 2021 年 8 月 12 日までの情報や新たに公開された研究を可能な限りアップデートして執筆したものである。COVID-19 の状況、政府の対応は刻一刻と変化することにご留意いただきたい。

第 1 節では、特措法に基づき採られた COVID-19 対策を紹介する。第 2 ・ 3 節は、COVID-19 対策に関連する、個人の自由を保障する概念及び個人の自由を制限しうる概念を指摘する。第 4 節では、上記の概念を COVID-19 対策に適用し、COVID-19 対策を検討する際の憲法的論点を整理する。

## I. 特措法と COVID-19 対策

2021 年 8 月現在、日本における COVID-19 対策の基盤となっているのは、特措法である。特措法は、2009 年に新型インフルエンザが世界的に流行し、医療資源が逼迫したことを契機として、2012 年に成立し、2013 年に施行された ^10^。2020 年に COVID-19 が特措法の対象となった後、2021 年に改正されている。

特措法の対象は新型インフルエンザ、再興型インフルエンザ及び新感染症である ^11^。ここで重要なのは、特措法の対象となる感染症は、国民の多くが免疫を獲得していないため、急速な蔓延により国民の生命及び健康に重大な影響を与える恐れがある点である ^12^。そのため、既知の感染症より強い措置が必要となる。

特措法の特徴は、緊急事態宣言の発出や外出自粛要請、施設の使用制限要請・指示等が可能になることだが、厚生労働省幹部によれば、日本で初めて COVID-19 感染者が確認された 2020 年 1 月時点では特措法の適用が必要であるとの認識は共有されておらず、感染症法及び検疫法を基盤とした対応が想定されていた ^13^。同年 2 月 1 日に、COVID-19 は感染症法に基づく二類相当の「指定感染症」とされ、感染の疑いがある患者の入院措置が可能になった ^14^。また同日、COVID-19 が検疫法上の「検疫感染症」となり、強制力を伴う入国時の診察・検査が可能になった ^15^。

しかし同年 2 月 21 日に国内感染者数が 100 人を超えると社会的な緊張が高まり、2 月 27 日には当時の安倍首相が、感染拡大抑制のために必要な法案を早急に準備するよう指示した ^16^。そして 3 月 13 日に特措法が改正され、翌 14 日より COVID-19 が特措法の対象となった ^17^。

2020 年 3 月から 2021 年 2 月まで適用されていた特措法では、二段階構造により新型感染症のまん延予防を試みた ^18^。第一段階は政府対策本部の設置である。従来のインフルエンザより症状が深刻である新型感染症が発生した際に、政府対策本部、都道府県対策本部が設置される ^19^。都道府県対策本部長となる都道府県知事は、特措法 24 条第 9 項に基づき、団体、個人に必要な協力を要請する事ができる。

第二段階が緊急事態宣言である。首相は、感染症が全国的なまん延により、国民社会、国民生活に甚大な影響を及ぼす場合に緊急事態宣言を発出し、緊急事態措置を実施すべき期間、区域を指定する ^20^。特措法は緊急事態宣言発出時の専門家の役割に触れていないものの、首相が専門家に諮問することが想定されている ^21^。緊急事態措置の対象となった地域では、都道府県知事が要請・指示等の主体となるが、政府が定める基本的対処方針 （特措法 18 条）に沿って要請・指示等を行うこととなる ^22^。また、新感染症対策であっても、国民の自由や権利への制限は最低限であるべきであると規定されている ^23^。

2021 年 2 月まで適用されていた特措法では、45 条に基づき、緊急事態措置の対象となった地域において、都道府県知事が不要不急の外出自粛要請及び施設使用制限の要請を行う事が可能となった ^24^。施設の使用制限要請に応じない場合、罰則を課すことはできないものの、知事が施設管理者に法的義務を課す指示を行う権限が与えられた ^25^。45 条に基づいて施設使用制限が要請・指示された際には公表されることとなっており ^26^、公表による社会的な影響も指摘された ^27^。

2020 年 3 月 14 日に COVID-19 が特措法の対象となると、同法 15 条に基づく政府対策本部が設置され ^28^、各都道府県知事が同法 24 条に依拠した団体・個人への要請を行うことが可能になった ^29^。感染確認者数の増加と社会的危機感の高まりを受け ^30^、2020 年 4 月 7 日から 5 月 25 日にかけて日本全国もしくは一部に緊急事態宣言が発出された ^31^。緊急事態宣言解除後も、同法 24 条第 9 項を根拠として、断続的に外出自粛要請を行った都道府県がある ^32^。また、2020 年 4 月の緊急事態宣言発出後は、解除後も含め、散発的に同法 24 条第 9 項を根拠として休業や営業時間短縮の要請を出した都道府県があった ^33^。

2020 年末から感染確認者数が 1 日に 3000 人を超える日が続き、重症者数も多かったため ^34^、2021 年 1 月 8 日から埼玉県、千葉県、東京都、神奈川県を対象として緊急事態宣言が発出された ^35^。しかし都道府県知事による要請に応じない事例も多く ^36^、感染状況の改善が見られなかったため、緊急事態宣言発出中の 2021 年 2 月 3 日に特措法が改正され、2 月 13 日より施行された。主な改正点は以下の 2 点である。第一に、「まん延防止等重点措置（以下、重点措置）」が導入された。これは、緊急事態宣言以前に効果的な感染症対策を実施可能にするための措置であり、従来の政府対策本部の設置と緊急事態宣言の間に位置付けられる。すなわち、従来の二段階から三段階で感染症に対応することとなった。重点措置が適用されると都道府県知事は、事業者に対して営業時間の変更を要請することができる ^37^。また住民に対して営業時間の変更の要請に従わない場への出入り自粛を要請することができる ^38^。事業者が営業時間の変更要請に従わない場合、都道府県知事は、営業時間の変更を命令することができる ^39^。重点措置の際には、休業の要請・命令が可能になる緊急事態宣言の前段階として、営業時間の変更の要請・命令が可能になる ^40^。

第二に、緊急事態宣言及び重点措置では、命令を出すことが可能になり ^41^、命令への違反行為には罰金を課すことができるようになった。緊急事態宣言時の違反には30万円までの過料 ^42^、重点措置時の違反には20万円までの過料を課すことができるようになった ^43^。特措法改正後も緊急事態宣言及び重点措置は複数の都道府県を対象として断続的に発出されている（ 表 1 参照。内閣官房ウェブサイト ( http://corona.go.jp/emergency/) を参考に著者が作成）。

**表1.** まん延防止等重点措置、緊急事態宣言の発出状況と適用/解除地域

まん延防止等重点措置			緊急事態宣言		
**2020年特措法**					
			**日付**	**適用/解除**	**適用/解除地域**
			2020年4月7日	適用	埼玉、千葉、東京、神奈川、大阪、兵庫、福岡
			2020年4月16日	追加適用	埼玉、千葉、東京、神奈川、大阪、兵庫、福岡を除く40道府県
			2020年5月14日	一部解除	北海道、埼玉、千葉、東京、神奈川、京都、大阪、兵庫を除く 39 県
			2020年5月21日	一部解除	京都、大阪、兵庫
			2020年5月25日	全国解除	北海道、埼玉、千葉、東京、神奈川
		
			2021年1月8日	適用	埼玉、千葉、東京、神奈川
			2021年1月14日	追加適用	栃木、岐阜、愛知、京都、大阪、兵庫、福岡
**2021年改正特措法**					
**日付**	**適用/解除**	**適用/解除地域**	**日付**	**適用/解除**	**適用/解除地域**
			2021年2月7日	一部解除	栃木
			2021年3月7日	一部解除	岐阜、愛知、京都、大阪、兵庫、福岡
			2021年3月21日	全国解除	埼玉、千葉、東京、神奈川
					
2021年4月5日	適用	宮城、大阪、兵庫			
2021年4月12日	追加適用	東京、京都、沖縄			
2021年4月20日	追加適用	埼玉、千葉、神奈川、愛知			
2021年4月25日	追加適用	愛媛			
2021年4月25日	宣言へ移行	東京、京都、大阪、兵庫	2021年4月25日	適用	東京、京都、大阪、兵庫
2021年5月9日	追加適用	北海道、岐阜、三重			
2021年5月11日	一部解除	宮城			
2021年5月11日	宣言へ移行	愛知			
			2021年5月12日	追加適用	愛知、福岡
2021年5月16日	宣言へ移行	北海道	2021年5月16日	追加適用	北海道、岡山、広島
2021年5月16日	追加適用	群馬、石川、熊本			
2021年5月22日	一部解除	愛媛			
2021年5月23日	宣言へ移行	沖縄	2021年5月23日	追加適用	沖縄
2021年6月13日	一部解除	群馬、石川、熊本			
2021年6月20日	一部解除	岐阜、三重	2021年6月20日	一部解除	北海道、東京、愛知、大阪、京都、兵庫、岡山、広島、福岡
2021年6月21日	宣言から移行	北海道、東京、愛知、京都、大阪、兵庫、福岡			
2021年7月11日	一部解除	北海道、愛知、京都、兵庫、福岡			
2021年7月11日	宣言へ移行	東京			
			2021年7月12日	追加適用	東京
2021年8月2日	宣言へ移行	神奈川、千葉、埼玉、大阪	2021年8月2日	追加適用	埼玉、千葉、神奈川、大阪
2021年8月2日	適用	北海道、石川、京都、兵庫、福岡			
2021年8月8日	追加適用	福島、茨城、栃木、群馬、静岡、愛知、滋賀、熊本			

内閣官房ウェブサイト( http://corona.go.jp/emergency/) を参考に著者が作成.

## II. 個人の自由を保障する概念

罰則の有無にかかわらず、特措法による休業や営業時間短縮要請・指示・命令、外出自粛要請は、国家権力による市民の自由の制限となりうる。特に 2021 年 2 月の特措法改正により導入された、罰則が課されうる休業や営業時間短縮の命令は、市民の自由制限の度合いが強い。国家による介入の回避を重要視する近代立憲主義を基盤とし ^44^、大日本帝国憲法下での国家権力の強大化を経験した後に制定された日本国憲法の視点からは、認められるべき市民の自由制限の程度が重要な論点となる。本節では、日本国憲法が保障する個人の自由として、営業の自由及び移動の自由についての判例や学説を検討する。

休業や営業時間短縮の要請・指示・命令は、営業の自由を制限しうる。営業の自由に関連する条文は、憲法 22 条、29 条、13 条である。営業の自由自体を保障する文言は憲法に存在しないが、判例、通説によれば憲法 22 条の職業選択の自由により営業の自由が保障されると考えられている ^45^。また、22 条の職業選択の自由に加えて 、29 条に規定される財産権により営業の自由が保障されるとの見解もある ^46^。その上で、憲法 13 条に規定される人格権と関連して営業の自由を位置付けることもできる。薬事法違憲判決は、職業が「個人の人格的価値とも不可分の関連を有する」としており ^47^、学説でも営業の自由と人格的価値の関連が指摘されている ^48^。人格的価値は憲法 13 条から導出できる人格権と関連しており ^49^、営業の自由が人格権の視点からも重要であることを示唆している。

外出自粛要請は、移動の自由を制限しうる。関連する条文は憲法 22 条及び 13 条である。移動の自由は、居住・移転の自由を規定する憲法 22 条に基づき保障されると考えられている ^50^。その上で、移動の自由は人の活動領域を拡大し、人格形成に寄与するとの指摘もあり ^51^、憲法 13 条を基盤とする人格権として移動の自由が捉えられる可能性もある ^52^。

## III. 個人の自由を制限しうる概念

憲法には個人の自由を保障する概念がある一方で、個人の自由を制限しうる概念も存在する。自由の行使が生命権、生存権・公衆衛生、公共の福祉の脅威となる場合、自由は一定の制限を受ける。

第一に、生命権の脅威となる自由は制限されうる。生命権の根拠は憲法 13 条である。日本国憲法における生命権は、1987 年に櫻田が提唱し ^53^、石村 ^54^、ウィリアムズ ^55^、小林 ^56^が議論を深め、山内が 2000 年に体系化を試みた ^57^。しかし通説では、憲法 13 条の「生命、自由及び幸福追求に対する国民の権利」が幸福追求権を意味するとされ ^58^、判例も憲法 13 条が生命権を規定するとの理解を示しておらず ^59^、生命権については十分議論されてこなかった ^60^。しかし、生命権は以下の二つの理由から、幸福追求権とは別個に理解されるべきである。第一に、条文の文理上の解釈である。生命に対する権利、自由に対する権利及び幸福追求に対する権利は並列で表記されているため、生命に対する権利は幸福追求に対する権利に包含されず、別個に位置づけられるべきである ^61^。第二に、権利の性質である。生命の維持は幸福追求権を含むあらゆる権利の前提であり、「もっとも基本的な人権」と考えられるべきである ^62^。生命への権利は、国王の圧政からの解放という近代憲法成立の文脈では、「国家に殺されない権利」が中心的な議論であり、主に国家の介入を否定する自由権的な文脈で議論されることがあった ^63^。しかし、COVID-19 への対応が必要となる今日においては、国家の積極的な介入を要求する社会権的な視点を含めて生命権を捉える必要があり、国家による生命の保護義務も含まれるべきである ^64^。COVID-19 による死者は 2021 年 8 月 14 日現在、日本で 15393人確認されており ^65^、COVID-19 が生命権の脅威となることを示している ^66^。よって、生命権保障の手段として COVID-19 対策が求められる。

第二に、憲法 25 条の生存権の保障・公衆衛生の保全を目的とした自由の制限は認められうる。COVID-19対策は公衆衛生の保全により実現するため、生命権保障のための手段として生存権保障・公衆衛生保全を位置付けることができる。しかし通説、判例によれば、憲法 25 条に基づく具体的請求権は認められておらず ^67^、感染症対策の請求権も認められないであろう。その理由として一般的に①不確定概念性 ^68^、②審査不適合性、③作為方法不特定性 ^69^、④予算随伴性が挙げられてきた ^70^。しかし、上記の具体的請求権を認めない理由は批判的に検討すべきである。①憲法上における生存権の文言が不明確であるとする不確定概念性については、例えば「わいせつ」概念が裁判所によって解釈されている事例があり、憲法25条における「健康で文化的な最低限度の生活」も裁判所が解釈することができる ^71^。また「一票の格差」問題においても裁判所が違憲審査を行っており、裁判所が基準を示すことは可能であるし、学説も解釈を行っている ^72^。②専門的・政策的な審査が必要とされるために司法審査になじまないとする審査不適合性については、非法的な判断に法的な審査を行うのが司法審査であり ^73^、法の視点から望ましい解釈を検討するのは司法や法学の役割と言える。③生存権実現の方法が憲法からは明らかでないとの作為方法不特定性については、法的な視点から作為方法を特定するのが困難であるにしても、作為方法を他の視点を導入して明らかにすることは可能である。COVID-19対策には公衆衛生の視点が必要であるが、専門家の見解を参照しつつ、憲法における生存権が保障されているかを法的に検討することは、可能でありかつ望ましい。④生存権保障のためには財政民主主義のもと立法府が策定する予算が必要であるため、司法のみで生存権保障のための判断はできないとする予算随伴性については、生存権保障のためには予算が必要な場合もあるものの、少なくとも生存権保障のために必要な手段を法的に検討する際には予算を考慮に入れるべきでない。また、例えば営業や移動の自由の制限など、必ずしも予算を必要としない手段による生存権の保障及び公衆衛生の保全も考えられる ^74^。さらに、予算への影響を検討することが「過剰な自己抑制」であり、「司法部の政治部門に対する介入になる」との痛烈な批判もなされている ^75^。よって、生存権の内容を法学・司法が具体化し、憲法を基盤とした具体的請求権が認められるべきである。なお、従来では生活保護などの給付請求権が議論されてきたが、生存権保障の方策は給付に限らない。憲法 25 条第 2 項は、「国は、すべての生活部面について、社会福祉、社会保障及び公衆衛生の向上及び増進に努めなければならない。」と規定しており、「公衆衛生の向上及び増進」のために必要な方策を請求することも、憲法 25 条に基づき可能なはずである。上記の請求がなされた際には、公衆衛生の保持を目的とした個人の自由の制限が正当化されうる。COVID-19対策として、営業や移動の自由の制限が有効であると考えられる際には、こうした措置を採ることが生存権の視点から求められる可能性がある。

第三に、憲法 13 条に規定される「公共の福祉」は、個人の自由を制限する根拠となる。同条は、国民の権利が「公共の福祉に反しない限り、（中略）最大の尊重を必要とする」と規定しており、公共の福祉に適合しない場合、自由・人権が制限されうる。判例は公衆浴場法事件や薬局距離制限事件等を通して、公共の福祉概念に国民の保健・環境衛生が含まれるとしてきた ^76^。また学説も、他者の権利・利益確保の文脈で、公衆衛生が公共の福祉に含まれるとしており ^77^、これは妥当な見解であろう。よって、公衆衛生の保全を目的とする COVID-19 対策は公共の福祉に含まれ、個人の自由の制限が正当化されうる。なお日本には、感染力が低いと認識されていたにもかかわらず、公共の福祉の名の下にハンセン病患者の隔離が行われた歴史があり ^78^、公共の福祉と感染症の関係性については慎重な検討が必要である。

## IV. COVID-19 対策への適用

以上では、日本国憲法における個人の自由を保障する概念及び個人の自由を制限しうる概念を紹介した。本節ではそれぞれの概念を COVID-19 対策に適用し、COVID-19 対策のための自由の制限が認められるかを検討する。

まず、営業の自由、移動の自由を制限しうる要請・指示・命令が憲法上認められるかを検討する。営業の自由及び移動の自由は、居住、移転及び職業選択の自由を規定する憲法 22 条、財産権を規定する 29 条、人格権を規定する 13 条が主に関連している。これらの権利は公共の福祉の制約を受けるが、権利の性質により制約の程度が異なる。判例、通説では、精神的自由についてはより厳格な基準で、経済的自由についてはより緩やかな基準で公共の福祉を捉える、「二重の基準」論が有力になっている ^79^。本稿では、試論的に従来の「二重の基準」論を発展させ、権利の性質でなく他者の人権の制約度合いを基盤として制約を検討する「二重の基準」論を採用する。制約度合いが小さい場合には厳格な基準で公共の福祉を捉え、他者の人権の制約度合いが大きい場合には緩やかな基準で公共の福祉を捉える。一般的に、精神的自由は他者の人権の制約度合いが小さく、経済的自由は他者の人権の制約度合いが大きい傾向にあると考えられるため、従来の「二重の基準」論と同様に、精神的自由についてはより厳格な基準で、経済的自由についてはより緩やかな基準で公共の福祉を捉える事になる。しかし、他者の人権の制約度合いが大きい場合には精神的自由であっても緩やかな基準で公共の福祉を捉え、他者の人権の制約度合いが小さい場合には経済的自由であっても厳格な基準で公共の福祉を捉えることで、人権保障のためのCOVID-19対策を検討することができる。

営業の自由及び移動の自由の根拠となる居住、移転及び職業選択の自由や財産権は経済的自由に含まれると考えられる ^80^。よって、緩やかな基準で公共の福祉を捉え、一定程度の営業の自由及び移動の自由の制約が許容される。

なお、営業の自由及び移動の自由の基盤を精神的自由である人格権の一部として位置付け、従来の「二重の基準」を適用すれば、公共の福祉がより厳格に捉えられ、COVID-19 対策よりもこれらの自由が優先されるとの議論もありうる。しかし、COVID-19 対策のための営業の自由及び移動の自由の制約は正当化されるべきである。営業の自由及び移動の自由が、基盤的な権利である生命権保障の障害となりうるからである。司法も、営業の自由と「人格的価値」との関係性を認めた薬事法違憲判決では、営業の自由と人格的価値としての関連性に言及した後に、職業は「社会的相互関連性が大きい」ため、「公権力による規制の要請がつよ」いとしている ^81^。これは精神的自由であっても社会的影響を十分に考慮すべきことを裁判所も認めていることを示す。また、COVID-19 の特性も検討する必要がある。従来、個人の移動による他者の人権の制約度合いについては十分に検討されてこなかった。しかし COVID-19 には無症状者が存在し、感染者が明らかでない。そのため、効果的な COVID-19 対策のためには、感染確認者のみでなく、市民一般を対象とした移動の制限が必要になる可能性がある。移動を制限するためには営業の自由への制限も効果的であろう。よって、COVID-19 対策としての営業の自由及び移動の自由の制限は許容される。

その上で、生命権、生存権・公衆衛生の視点を基盤とすれば、COVID-19 による生命へのリスクを減らす方策を国家が採る必要がある。特に生命権は人権の基盤であるため、本質的にその保障が最も重要である。よって、自由を制限したとしても公衆衛生の保全による生命権、生存権の保障が必要である。2021 年 8 月時点で新型コロナウイルス感染症対策分科会は、特に感染拡大が懸念される東京都に関して、同年7 月前半に比べて人出を半減するように要請する提言を行った ^82^。COVID-19 に有効なワクチン接種を加速化させることも生命権保障のためには重要であるが、変異ウイルスにはワクチンの効果が低く、重症化のリスクがあるとの指摘もあることから ^83^、COVID-19 対策には休業や営業時間短縮の要請・命令及び外出自粛要請・外出制限等の措置が必要となるであろう ^84^。2021 年 8 月時点で生命権への脅威となる感染拡大が止まらない中、科学的知見に依拠したさらに有効な手段による感染拡大の予防が求められている。

最後に、罰則を伴う措置について補完的に検討する。本研究の主眼ではないため、詳細は今後の研究課題とするが、論点整理のための検討を行う。2021 年 2 月の特措法改正以降、緊急事態宣言もしくは重点措置の際に、休業や営業時間短縮の命令を違反した者には過料を課すことが可能になったが、日本国憲法は罰則のある外出制限、休業や営業時間短縮の措置を許容していると考えられる ^85^。COVID-19 対策が生命権保障のために有効であり、公共の福祉に適合すれば、罰則を禁止する根拠を見いだすことは困難だからである。よって、例えば都市封鎖も現行憲法において可能であると言える。とはいえ、罰則のある外出制限、休業や営業時間短縮の措置が生命権保障のために必要な状況でなければ、憲法はこれらの措置を求めているわけでもなく ^86^、罰則の有無は立法府の判断に委ねられている。罰則を導入する際には、行政法における比例原則を考慮する必要がある。比例原則の内容として、目的適合性の原則、必要性の原則、狭義の比例性の原則が挙げられる ^87^。よって罰則は必要最小限で、かつ法益に与える影響が不釣り合いであってはならない ^88^。2021年8月時点では、まん延防止等重点措置の際には営業時間短縮の命令に違反した場合に20万円の過料、緊急事態宣言の際には休業の命令に違反した場合に30万円の過料が課せられうる。この比例原則に照らした評価は今後の課題としたい。

## おわりに

本稿は、COVID-19 対策に関わる憲法上の論点を「個人の自由を保障する概念」及び「個人の自由を制限しうる概念」に整理し直した上で、COVID-19 対策が公共の福祉に適合すると捉えられ、特措法に基づく休業や営業時間短縮の要請・命令、外出自粛要請に憲法上の制約はないと論じた。その上で、生命権・生存権の議論を基盤として、国家による積極的な COVID-19 対策が求められると述べた。また、比例原則を考慮する必要はあるものの、憲法は罰則のある措置を許容しているとまとめた。

最後に今後の検討課題として、2021 年 2 月に導入された罰則の意義と、ポスト・コロナ時代の国家観と憲法を挙げる。第一に、罰則導入の意義についてである。2020 年 12 月には、営業時間短縮の要請を遵守する事業者が限定されているとの報告があり、特措法改正時の論点は、罰則導入により休業もしくは営業時間の短縮が徹底されるか、であった ^89^。しかし、2021年8月の現状を踏まえると、罰則の有無以上に、社会による政府の対策の受け止め方が対策の実効性確保のために重要であると考えられる。2021 年 8 月時点で発出されている緊急事態宣言や重点措置の効果についてNHKが実施した調査では、69%が「あまりない」もしくは「まったくない」と回答した ^90^。効果がないとの評価は、緊急事態宣言や重点措置の際の要請や命令への罰則が不十分であるという認識による可能性もあるが、政府の対策への社会的信頼が失われている可能性も指摘できる ^91^。実効性を確保できない場合には罰則の意義は薄れ、今後政府への社会的信頼を取り戻すことも、有効な対策のために求められる。

第二に、ポスト・コロナ時代の国家観と憲法について検討する必要がある。従来日本の憲法学は、大日本帝国憲法下の経験から自由の制限に抑制的な立場をとってきた ^92^。また特措法も、2012 年の成立以降 2021 年 2 月の改正に至るまで、罰則規定が不在であった。これらの前提には、国家の権限強化への警戒感がある ^93^。日本国憲法及びその解釈を行う憲法学は第二次世界大戦の経験を踏まえ、他国等の「敵」を想定せず、国家の権限強化に抑制的だったのである ^94^。しかし COVID-19 は、この国家観に変化をもたらす可能性がある。感染症という自然環境の影響がある問題に直面している中、生命権保障のためには国家による積極的な COVID-19 対策が求められる。また、国民が国家の権限強化を求める動きがあることも指摘すべきである。2020 年 6 月に NHK が実施した世論調査では、62% が外出禁止や休業を強制できる法改正が必要であると回答した ^95^。また 2021 年 1 月に NHK が実施した世論調査では、48% が特措法への罰則の明記に賛成と回答しており ^96^、罰則のある措置に肯定的な意見が一定数あることを示している ^97^。政府への社会的信頼が下がっていると考えられる2021年8月時点での世論は不明であるが、感染症が社会の脅威になることで、国家への権限強化を求める見解が出ていることは重要な点であろう。国家に強力な権限を認めることには全体主義につながるとの懸念があり ^98^、国民が国家による罰則を望む現象が、近代立憲主義の視点から危険視されるのは当然である ^99^。しかし、新型コロナウイルスを含む新興の人獣共通感染症は増加傾向にあると指摘されており ^100^、今後も日本社会は感染症と向き合う必要がある ^101^。また、今後気象の極端現象が頻繁に発生するとの指摘があり ^102^、自然環境に対する社会の向き合い方も問われている。全体主義の歴史に学び、政治による恣意的な権力の集中を避けつつ、国家が生命権を保障し自然環境という「敵」の脅威に対応するために必要な措置を検討する必要がある。重要なのは、日本国憲法が従来想定してこなかった感染症や自然環境の問題が日本社会への脅威となっていることである。COVID-19 等感染症対策における「敵」はウイルスであり、科学的な専門知により根拠づけられる ^103^。憲法（学）が必ずしも想定してこなかった新たな、かつ人間社会に与える影響が巨大な「敵」が出現する中で、憲法（学）が描く国家観を今後再検討する必要がある ^104^。

COVID-19 は、社会に大規模かつ長期化する感染症と向き合うことを迫っており、日本国憲法にとって新たな課題である。憲法の枠組みに基づき法的な検討を行うとともに、日本社会の特性も考慮した有効な感染症対策により生命権を保障する、新たな国家観を構想していく必要がある。

## Footnotes

^1^
Daniel P. Oran, Eric J. Topol, “Prevalence of Asymptomatic SARS-CoV-2 Infection A Narrative Review.” *Annals of Internal Medicine*, 173: 362-367 (2020). DOI: 10.7326/M20-3012.

^2^
See, Hannah Ritchie, Esteban Ortiz-Ospina, Diana Beltekian, Edouard Mathieu, Joe Hasell, Bobbie Macdonald, Charlie Giattino, and Max Roser, “Policy Responses to the Coronavirus Pandemic,” OurWorldInData.org, accessed on 17 August 2021, https://ourworldindata.org/policy-responses-covid.

^3^
後述の江藤論文以外に、日本国憲法における「公共の福祉」の概念により、強制力のある COVID-19 対策が可能であるとの議論はあるが （井上達夫「コロナ・ラプソディー― パンデミックが暴く『無責任の体系』」法と哲学 6 号（2020 年） 1-43頁、36-37 頁。Lawrence Repeta, “The Coronavirus and Japan’s Constitution: Article 41 Provides the Government with Sufficient Power to Take Aggressive Action,” *The Japan Times*, 14 April 2020, https://www.japantimes.co.jp/opinion/2020/04/14/commentary/japan-commentary/coronavirus-japans-constitution/ (accessed on 10 January 2021).）、憲法上の人権に関連する概念を包括的に検討したものでない。なお、2020 年に公刊された COVID-19 対策を憲法の視点から検討した論考として、本稿内で特に言及するものに加え、以下が挙げられる。大林啓吾「感染症リスクと憲法 新型コロナウイルス流行を素材にして」小山剛・新井誠・横大道聡（編）『日常のなかの⟨自由と安全⟩生活安全をめぐる法・政策・実務』（弘文堂、2020 年） 410-426 頁、林知更「法律時評 憲法・非常事態・コロナ」法律時報 92 巻 13 号（2020 年） 1-3 頁。木村草太「休業・休校要請の法的根拠について 特措法 24 条第 9 項と 45 条第 2 項の関係（コロナ禍社会における法的諸問題(3)）」判例時報 2459 号（2020 年） 157-158 頁。曽我部真裕 「立憲主義のあり方から見る「自粛か強制か」問題」判例時報 2458 号（2020 年）144 頁。小山剛「自粛・補償・公表 インフォーマルな規制手法（コロナ禍社会における法的諸問題）」判例時報 2460 号（2020 年）145-146 頁。第1版脱稿後にも関連する論文・書籍が発表されている。統治機構を含めて、COVID-19 対策と立憲主義について包括的に検討した論文は以下がある。宍戸常寿「新型コロナウイルス感染症と立憲主義」法律時報 93 巻 3 号（2021 年）82-86 頁。COVID-19 を素材として憲法における感染症対策をまとめた書籍が以下である。大林啓吾（編）『感染症と憲法』（青林書院、2021 年）。

^4^
江藤祥平「匿名の権力――感染症と憲法」法律時報 92 巻 9 号 （2020 年） 70-77 頁。なお、数少ない COVID-19 対策に関する憲法的な論点を包括的に扱った論文として、以下も挙げられる。飯島滋明「コロナ感染症対策をめぐる憲法問題」名古屋学院大学論集　社会科学篇 57 巻 2 号（2020 年） 1-22 頁。しかし、公衆衛生保全の重要性や生命権については議論が十分になされていない。

^5^
第 1 版脱稿後に、医療体制に着目して COVID-19 と生命権の関係性について議論した論考として、以下が挙げられる。建石真公子「新型コロナウィルスと立憲主義-生命権・健康権と公益」憲法問題 32 号（2021 年） 91-104 頁。

^6^
江藤・前掲注 4 、 70 頁。

^7^
例えば芦部は「基本的人権の限界」と題した章の一部に「人権と公共の福祉」の節を置いており、人権と公共の福祉が対比されている。芦部信喜 （高橋和之補訂） 『憲法〔第 7 版〕』 （岩波書店、2019 年） 99 頁。

^8^
本稿では深く立ち入らないが、公共の福祉をめぐるパターナリズムによる制約の論点も関連する。パターナリズムによる制約については以下を参照。佐藤幸治『日本国憲法論〔第 2 版〕』 （成文堂、2020 年） 154頁。渋谷秀樹『憲法〔第 3 版〕』 （有斐閣、2019 年） 174-176 頁。

^9^
感染症法に関する議論は、太田匡彦「新型コロナウイルス感染症にテストされる感染症法 （上） 」法律時報 92 巻 9 号 （2020 年） 85-91 頁、太田匡彦「新型コロナウイルス感染症にテストされる感染症法 （下） 」法律時報 92 巻 11 号 （2020 年） 84-90 頁を参照。

^10^
新型インフルエンザ等対策研究会『逐条解説　新型インフルエンザ等対策特別措置法』 （中央法規出版、2013 年） 3-6 頁。なお本書籍は、COVID-19 の感染拡大を受け、中央法規出版ウェブサイト内において期間限定で無料公開されている。無料公開してくださった中央法規出版に感謝申し上げる。

^11^
新型インフルエンザとは、新たに人から人に伝染する能力を有することとなったウイルスを病原体とするインフルエンザを指す。再興型インフルエンザとは、かつて世界規模で流行したが、その後長期間流行しなかったインフルエンザで、再興したものを指す。新感染症とは、人から人に伝染する未知の疾病のうち、以下の三要件を満たすものをいう。第一に、当該疾病にかかった際の病状の程度が重篤であるもの、第二に、当該疾病のまん延が国民の生命、健康に重大な影響を与える恐れがあるもの、第三に、全国的かつ急速なまん延の恐れがあるもの、である。特措法 2 条第 1 号、感染症法 6 条第 7 項、同条第 9 項参照。なお、感染症法は既知の感染症も対象としている点が、特措法との違いである。

^12^
特措法 1 条。

^13^
一般社団法人アジア・パシフィック・イニシアティブ『新型コロナ対応民間臨時調査会調査・検証報告書』 （ディスカヴァー・トゥエンティワン、2020 年） 133-134 頁。

^14^
同書 134 頁。感染症法 19 条、20 条、26 条。

^15^
同書 134 頁。検疫法 13 条第 1 項。

^16^
首相官邸「令和 2 年 2 月 27 日　新型コロナウイルス感染症対策本部 （第 15 回） 」 https://www.kantei.go.jp/jp/98_abe/actions/202002/27corona.html （2020 年 2 月 27 日、2021 年 1 月 2 日アクセス）。

^17^
当時の経緯については、一般社団法人アジア・パシフィック・イニシアティブ・前掲注 13 、134-137 頁も参照。

^18^
新型インフルエンザ等対策研究会・前掲注 10 、8 頁。

^19^
特措法 15 条、22 条。

^20^
特措法 32 条。

^21^
新型インフルエンザ等対策研究会・前掲注 10 、117-118 頁。

^22^
都道府県知事が政府の基本的対処方針に基づく「総合調整」 （特措法 20 条第 1 項） に従わない場合、政府は「必要な指示」 （同法 33 条第 1 項） を出すことができる。

^23^
特措法 5 条。

^24^
2020年特措法 45 条第 1 ・ 2 項。この「要請」は不利益処分でなく、行政手続法 2 条第 1 項第 6 号に規定される「行政指導」と考えられる。内閣官房新型コロナウイルス感染症対策推進室長「事務連絡　第 45 条の規定に基づく要請、指示及び公表について」 （令和 2 年 4 月 23 日） https://corona.go.jp/news/pdf/yousei_shiji_0423.pdf （2021 年 1 月10 日アクセス） 。よって、法的義務は課されないと考えられている。参照、髙木光・常岡孝好・須田守『条解行政手続法』 （弘文堂、2017 年） 30 頁。また緊急事態措置の対象となった都道府県知事は、臨時の医療施設の開設のために私有地を使用することができる。特措法 48 ・ 49 条。

^25^
2020 年特措法 45 条第 3 項。参照、内閣官房新型コロナウイルス感染症対策推進室長・前掲注 24。

^26^
2020 年特措法 45 条第 4 項。

^27^
緊急事態宣言が発出された 2020 年 4 月には、休業もしくは営業時間短縮の要請を遵守していないと思われる店舗を私人が攻撃する、「コロナ自警団」や「自粛警察」と呼ばれる社会的制裁が横行した。参照、鴻上尚史・佐藤直樹『同調圧力:日本社会はなぜ息苦しいのか』 （講談社、2020 年） 128-132 頁。なお、2021 年 2 月改正前の特措法に関して片山は、政府対策本部が設置され、緊急事態宣言が発出されていない際に適用される特措法 24 条は、医師会や看護協会、大学の感染症研究者の協力を得るような事態を想定しており、営業停止などを要請する根拠とはならないとしていた。片山善博「連載 128 　片山善博の『日本を診る』『緊急事態宣言』をめぐる法律のとんでもない読み間違い」世界 2020 年 7 月 （934） 号 （2020 年） 105-106 頁。片山の主張するように、政府対策本部が設置され緊急事態宣言が発出されていない時は「身構える段階」であり、緊急事態宣言発出時とは性質が異なったと考えられる。しかし、同法 24 条に基づく要請は公表の規定がない一方で、緊急事態宣言発出時に適用される同法 45 条に基づく要請・指示は公表を義務付けていた。重要なのは、片山も指摘するように、改正前の2020年特措法において、45 条に基づく要請・指示を遵守しない場合に公表されたのではなく、要請・指示が行われた時点で公表された点である。同法 24 条に基づいて休業や営業時間短縮の要請が行われないにも関わらず、45 条に基づいて休業や営業短縮要請が行われた途端に公表されるのは、不合理である。そのため、24 条によっても休業や営業時間短縮の要請が可能であり、緊急事態宣言により公表が義務付けられていたと考える方が自然であろう。後述の通り、2021年2月の特措法改正によってまん延防止等重点措置が導入され、まん延防止等重点措置、緊急事態宣言下における要請・命令の際には公表すべきか否かを都道府県知事が判断できるようになった。また同措置や緊急事態宣言の際に罰則を課すことが可能になった。特措法改正後もまん延防止等重点措置、緊急事態宣言の際には罰則や公表が可能になるため、改正前の特措法における理由と同様に、24 条によっても休業や営業時間短縮の要請が可能であると解するのが合理的であろう。

^28^
2020 年 1 月 30 日に閣議決定により新型コロナウイルス感染症対策本部が設置され （「新型コロナウイルス感染症対策本部の設置について」 （令和 2 年 1 月 30 日閣議決定） https://www.cas.go.jp/jp/influenza/konkyo_corona.pdf （2021 年 1 月 3 日アクセス）。） 、同年 3 月 14 日に特措法に基づく政府対策本部となった。

^29^
同月には大阪府や東京都、神奈川県、埼玉県、千葉県、山梨県等が外出自粛を要請した 。nippon.com 「新型コロナウイルス感染症流行・ 3 月の主な動き」 https://www.nippon.com/ja/japan-data/h00730/ （2020 年 5 月 21 日、2021 年 1 月 4 日アクセス）。

^30^
一般社団法人アジア・パシフィック・イニシアティブ・前掲注 13 、141 頁。

^31^
2020 年 4 月から 5 月にかけての緊急事態措置については、以下を参照。内閣官房新型コロナウイルス感染症対策推進室「新型コロナウイルス感染症 緊急事態宣言の実施状況に関する報告」 （令和 2 年 6 月） 3 頁、 https://corona.go.jp/news/pdf/kinkyujitaisengen_houkoku0604.pdf （2021 年 1 月 3 日アクセス）。

^32^
例えば東京都は、緊急事態宣言解除後の 2020 年 7 月に不要不急の外出自粛要請を行った。 nippon.com 「新型コロナウイルス感染症流行・ 7 月の主な動き」 https://www.nippon.com/ja/japan-data/h00870/ （2020 年 11 月 19 日、2021 年 1 月 4 日アクセス）。

^33^
例えば東京都は緊急事態宣言解除後も休業要請を継続し、段階的に要請を緩和することとした。東京都「新型コロナウイルス感染症を乗り越えるためのロードマップ〜「新しい日常」が定着した社会の構築に向けて〜」 https://www.metro.tokyo.lg.jp/tosei/hodohappyo/press/2020/05/22/documents/11_00_1.pdf （2021 年 1 月 4 日アクセス） 。また大阪府は 2020 年 8 月に、府の指定した対策を講じない接待を伴う飲食店やカラオケ店に休業を要請し、それ以外の飲食店にも営業時間短縮を要請した。 nippon.com ・前掲注 32 。

^34^
首相官邸「令和 3 年 1 月 4 日　菅内閣総理大臣記者会見」 https://www.kantei.go.jp/jp/99_suga/statement/2021/0104kaiken.html （2021 年 1 月 4 日、2021 年 1 月 4 日アクセス）。

^35^
内閣官房新型コロナウイルス感染症対策推進室「新型コロナウイルス感染症緊急事態宣言」 https://corona.go.jp/emergency/ （2021 年 1 月 10 日アクセス）。

^36^
2020 年 12 月に神奈川県知事は、営業時間短縮の要請に 2 割しか応じていないとの県職員の感触を紹介している。東京新聞「時短営業『 2 割しか応じていない』と危機感　神奈川県・黒岩知事、根拠は『県職員が街を見回った実感』」 https://www.tokyo-np.co.jp/article/75931 （2020年 12 月 22 日、2021 年 1 月 10 日アクセス） 。こうした要請への遵守が不十分であるとの理解が特措法改正の契機になったと考えられる。

^37^
特措法 31 条の 6 第 1 項。

^38^
特措法 31 条の 6 第 2 項。

^39^
特措法 31 条の 6 第 3 項。

^40^
なお、対策本部が設置されていれば休業や営業時間短縮の要請は可能であると考えられるが、命令は重点措置や緊急事態宣言下でなければ出すことができない。注 27 参照。

^41^
特措法 31 条の 6 第 3 項、 45 条第 3 項。

^42^
特措法 79 条。

^43^
特措法 80 条。なお、罰則は常に課されるわけではない。

^44^
近代立憲主義については、長谷部恭男『憲法講話―― 24 の入門講義』 （有斐閣、2020 年） 1-14 頁を参照。

^45^
最高裁判所大法廷判決昭和 47 年 11 月 22 日刑集 26 巻 9 号 586 頁。芦部・前掲注7、 233 頁。佐藤・前掲注8、 335 頁。長谷部恭男『憲法〔第 7 版〕』 （新世社、2018 年） 241 頁。

^46^
浦部法穂『憲法学教室〔第 3 版〕』 （日本評論社、2016 年） 238-239 頁。渋谷・前掲注8、 295 頁。なお後述の通り、職業選択の自由及び財産権はともに経済的社会権であり、営業の自由の根拠が 22 条のみであっても、22 条及び 29 条であっても、本質的な違いはあまりないと思われる。よって本稿では、採るべき説についての議論は行わない。同様の見解は野中俊彦・中村睦男・高橋和之・高見勝利『憲法I〔第 5 版〕』 （有斐閣、2012 年） 472 頁を参照。

^47^
最高裁判所大法廷判決昭和 50 年 4 月 30 日民集 29 巻 4 号 572 頁。

^48^
佐藤・前掲注 8 、335 頁。江藤・前掲注 4 、74 頁。

^49^
憲法 13 条と人格権の関連については、渋谷・前掲注 8 、183 頁、野中・中村・高橋・高見・前掲注 46 、269-273 頁を参照。

^50^
浦部・前掲注 46、243 頁。野中・中村・高橋・高見・前掲注 46 、459 頁。

^51^
浦部・前掲注 46 、243 頁。野中・中村・高橋・高見・前掲注 46 、458 頁。

^52^
移動の自由を人身の自由（憲法31条）と関連させてから捉える可能性とその限界については以下を参照。曽我部真裕「日本国憲法における移動の自由」法学セミナー 798 号 （2021 年） 6-13 頁、8-9頁。

^53^
櫻田誉「憲法における生命権の保障―救命救急医療との関連を中心として―」関西大学法学論集 37 巻 2 ・ 3 号 （1987 年） 4 頁。

^54^
石村修「基本権の体系における生きる権利の意味」法学新報 96 巻 11 ・ 12 号 （1990 年） 98-107 頁。

^55^
Noel Williams, *The Right to Life in Japan* （London and New York: Routledge, 1997）.

^56^
小林直樹「人権価値を根底から考える――哲学的人間学の視点から」憲法問題 9 号 （1998 年） 156-159 頁。

^57^
山内敏弘「基本的人権としての生命権の再構成」杉原泰雄先生古稀記念論文集刊行会（編）『二一世紀の立憲主義―現代憲法の歴史と課題―』 （頸草書房、2000 年） 325-345 頁。加筆を経て、山内敏弘『人権・主権・平和――生命権からの憲法的省察――』 （日本評論社、2003 年） 2-32 頁にも収録されている。生命権論のその後の展開については、山内敏弘「基本的人権としての生命権の再定位」龍谷法学 53 巻 2 号 (2020 年) 43 頁を参照。

^58^
芦部・前掲注 7 、120 頁。佐藤・前掲注 8 、196 頁。野中・中村・高橋・高見・前掲注 46 、272 頁。浦部・前掲注 46 、45 頁。

^59^
判例では、憲法 13 条に基礎付けられる権利・自由として、私生活上の自由 （最高裁判所大法廷判決昭和 44 年 12 月 24 日刑集 23 巻 12 号1625 頁） と人格権 （最高裁判所大法廷判決昭和 61 年 6 月民集 40 巻 4 号 872 頁） が指摘されている。渋谷・前掲注 8 、183-184 頁。

^60^
山内・前掲注 57 （「基本的人権としての生命権の再定位」） 、1 頁。なお、詳細には論じられていないものの、辻村や渋谷が生命権の議論に触れており、生命権について議論されつつある。辻村みよ子『憲法〔第 6 版〕』 （日本評論社、2018 年） 140 頁。渋谷・前掲注 8 、185-187 頁。

^61^
山内・前掲注 57 （『人権・主権・平和――生命権からの憲法的省察』） 、4 頁。

^62^
同書、4 頁。

^63^
例えば、生田勝義「死刑と生命権 (再論) ――死刑憲法 13 条違憲論は死刑廃止に役立つか――」立命館法学 365 号 (2016 年) 110-141 頁は死刑の観点から生命権を検討している。

^64^
国家による保護義務を認めることは、国家の不当な介入を招く恐れが大きいとの批判もある。芦部は国家による介入の限定が困難であるとして、日本国憲法の解釈に国家の保護義務を導入することへの懸念を示している。芦部信喜『宗教・人権・憲法学』 （有斐閣、1999 年） 230 頁。しかし、生命の保護が人権保障の基盤であることを考えると、生命保護のための国家による積極的行動は必要であろう。また山内は、近代憲法の思想的基盤であるロックの社会契約論から、国家の市民の保護義務を導き出すことができると述べる。山内・前掲注 57 （「基本的人権としての生命権の再定位」） 、25-26 頁。なお、生命権の議論の先駆者と言える櫻田誉は、日本における救命救急医療体制が不十分であるとの認識から生命権の議論を行っており、COVID-19 対策に関する生命権の議論にも示唆的である。櫻田・前掲注 53 、254-273 頁。

^65^
厚生労働省「データからわかる―新型コロナウイルス感染症情報―」 https://covid19.mhlw.go.jp/extensions/public/index.html （2021 年 8 月 15 日アクセス）。

^66^
根岸も、日本政府が生命を保護する義務を十分に果たしていないと評価している。 Yota Negishi, “Japan,” Bonavero Institute of Human Rights, *Bonavero Report 7/2020: A Human Rights and Rule of Law Assessment of Legislative and Regulatory Responses to the COVID-19 Pandemic across 27 Jurisdictions* (2020), https://www.law.ox.ac.uk/sites/files/oxlaw/bonavero_report_72020_updated.pdf (accessed on 17 August 2021), 277. ただ、例えば日本における癌による死者は 2018 年に 373,584 人であり （国立がん研究センター 癌情報サービス「最新がん統計」 https://ganjoho.jp/reg_stat/statistics/stat/summary.html （2020 年 7 月 6 日、2021 年 1 月 5 日アクセス。）） 、COVID-19 が他の要因と比較して圧倒的な数の死者の要因となっているわけではないことには留意が必要である。何が生命権の侵害となるかとの見解には、社会的価値判断が必要であろう。本稿は、COVID-19 が感染症であり、一人の感染が他者の生命にも影響を及ぼす可能性がある点、COVID-19 対応のために医療体制が逼迫している点を踏まえ (NHK 「“災害時の状況に近い 医療ひっ迫” 新型コロナ 専門家会合」 https://www3.nhk.or.jp/news/special/coronavirus/medical/detail/detail_132.html(2021 年 8 月 11 日、2021 年 8 月 15 日アクセス) 。) 、COVID-19 が生命権への脅威であると論じる。

^67^
通説は、芦部・前掲注 7 、120 頁、浦部・前掲注 46 、250-251 頁を参照。判例は、憲法 25 条が「すべての国民が健康で文化的な最低限度の生活を営み得るように国政を運営すべきことを国の責務として宣言したにとどまり、直接個々の国民に対して具体的権利を賦与したものではない」とする。最高裁判所大法廷判決昭和 42 年 5 月 24 日民集 21 巻 5 号 1043頁。

^68^
芦部・前掲注 7 、279 頁。

^69^
浦部・前掲注 46 、251 頁。

^70^
棟居快行「生存権の具体的権利性」長谷部恭男（編）『リーディングズ現代の憲法』 （日本評論社、1995 年） 156-166 頁。

^71^
同論文、159 頁。

^72^
これまでの主な判例については芦部・前掲注 7 、146-153 頁を参照。また学説でも、投票価値が二対一以上になることは法の下の平等の観点から違憲であるとの見解が示されている。芦部・前掲注 7 、145 頁。

^73^
棟居・前掲注 70、160 頁。

^74^
例えば営業や移動の自由を制限することによる生存権保障及び公衆衛生の保全は、必ずしも予算が必要な措置ではないだろう。

^75^
渋谷・前掲注 8 、278 頁。

^76^
渋谷秀樹「『公共の福祉』とは何か」岡田信弘・笹田栄司・長谷部恭男（編）『憲法の基底と憲法論――思想・制度・運用――』（信山社、2015 年） 45 頁。

^77^
同論文、55-56 頁。

^78^
参照、財団法人日弁連法務研究財団 ハンセン病問題に関する検証会議『ハンセン病問題に関する検証会議 最終報告書』 (2005 年) 129 頁、 https://www.mhlw.go.jp/topics/bukyoku/kenkou/hansen/kanren/4a.html （2021 年 1 月 10 日アクセス） 。

^79^
芦部・前掲注 7 、104-107 頁。最高裁判所大法廷判決昭和 50 年 4 月 30 日民集 29 巻 4 号 572 頁。

^80^
芦部・前掲注 7 、233-250 頁。

^81^
最高裁判所大法廷判決昭和 50 年 4 月 30 日民集 29 巻 4 号 572 頁。

^82^
新型コロナウイルス感染症対策分科会 「期間限定の緊急事態措置の更なる強化に関する提言 令和 3 年 8 月 12 日（木） 」 https://www.cas.go.jp/jp/seisaku/ful/taisakusuisin/bunkakai/dai5/kyouka_teigen.pdf（2021 年 8 月 12 日、2021 年 8 月 17 日アクセス）。

^83^
Rachel Herlihy, Wendy Bamberg, Alexis Burakoff, *et al*. “Rapid Increase in Circulation of the SARS-CoV-2 B.1.617.2 (Delta) Variant — Mesa County, Colorado, April–June 2021.” *MMWR Morb Mortal Weekly Report*, 70: 1084-1087 (2021). DOI: 10.15585/mmwr.mm7032e2.

^84^
ただ外出制限を課す場合でも、ドメスティック・バイオレンスの被害者にとっては外出制限が命の危険につながる可能性もある。こうしたことも踏まえ、生命権へのリスクを考慮に入れつつ外出制限の可能性を検討する必要がある。なお本稿では詳細に立ち入らないが、科学的に望ましい対処法が明らかでない場合の対策も重要である。新型コロナウイルスは変異することもあり、その全貌は明らかでないからである。こうした場合には、国際的に環境問題等に用いられる「予防原則 (precautionary principle) 」を導入することも、生命権を保障するために検討する必要があろう。予防原則とは、「科学的知見が十分に備わっていなくても、ひとたび問題が生じたとき、深刻かつ不可逆的な被害が発生する場合に、何もしないのではなく、必要な対策をとるべきである、とする法原則」である。松本和彦「公法解釈における諸原理・原則の対抗――憲法学から見た比例原則・予防原則・平等原則」公法研究 81 号 （2019 年） 67 頁。予防原則については批判もあるが（参照、西原博史「リスク社会・予防原則・比例原則」ジュリスト 1356 号 （2008 年） 75 頁。） 、生命が人権の基盤であり、一度失われると回復不可能であることを考えると、予防原則を採る必要がある。感染リスクがないことが立証されるまで、感染リスクとなりうる行為の積極的な抑制を検討する必要があろう。COVID-19 対策をめぐる予防原則と憲法学説の関係性については以下を参照。尾形健「新型コロナと法（第 5 回）「新型コロナウイルス禍」の福祉国家 : 憲法研究者からみた「新型コロナと法」」法学セミナー 790 号（2020 年） 56-61 頁、60 頁。

^85^
曽我部も、強制力のある外出制限が一概に憲法違反になるとは言えないとの見解を示している。曽我部真裕「日本国憲法における移動の自由」法学セミナー 798 号（2021 年） 6-13 頁、13 頁。

^86^
しかし、生命権保障のために必要な状況になれば、罰則を課す休業や営業時間短縮の措置が憲法の視点から求められる可能性があることには留意が必要である。

^87^
高橋明男「比例原則審査の可能性」法律時報 85 巻 2 号 （2013 年） 21 頁。憲法訴訟における比例原則については以下を参照。松本・前掲注 84 、63-66 頁。

^88^
先述の予防原則と比例原則と緊張関係を孕む可能性があるが、措置自体については予防原則を検討する必要がある一方で、罰則については比例原則を適用することが望ましいと考えられる。措置と罰則は手段が異なるため、異なる原則を適用することが可能であろう。

^89^
2020 年 5 月の時点で江島は、特措法改正前の自粛要請による対策が、政府に強大な権限を与えずとも感染拡大を予防できるか否かを占う試金石になると述べていた。 Akiko Ejima, “Japan’s Soft State of Emergency: Social Pressure Instead of Legal Penalty,” in *Verfassungsblog on Matters Constitutional*, 13 May 2020, https://verfassungsblog.de/japans-soft-state-of-emergency-social-pressure-instead-of-legal-penalty/ (accessed on 10 January 2021). なお、2020 年 4 月に初めて緊急事態宣言が発出された際には、休業もしくは営業時間短縮の要請を遵守していないと思われる店舗を私人が攻撃する、「コロナ自警団」や「自粛警察」と呼ばれる社会的制裁が横行した。2021 年 2 月の特措法改正時には、罰則の導入により社会的制裁が減少するかとの論点もあった。しかし政府の発信への信頼が揺らいでいると考えられ、そもそも罰則の有無に関わらず社会的制裁の度合いが低いと思われる 2021 年 8 月現在では、重要な論点とは言えないであろう。社会的制裁を減少させるために罰則を導入すべきだとの議論への批判は以下を参照。曽我部真裕「立憲主義のあり方から見る「自粛か強制か」問題」法律時報2458号、144頁。

^90^
NHK「緊急事態宣言と措置の効果「あまりない」49%」 https://www.nhk.or.jp/politics/articles/lastweek/65491.html （2021 年 8 月 10 日、2021 年 8 月 14 日アクセス）。

^91^
2021 年7月に東京オリンピックが開催され、そのために外出自粛要請を遵守する機運が低下したとの指摘もある。原田隆之「感染爆発なのに危機感がない　人々の心理に何が起こっているのか　効果的な対策は?」 https://news.yahoo.co.jp/byline/haradatakayuki/20210810-00252430 （2021 年 8 月 10 日、 2021 年 8 月 14 日アクセス）。

^92^
日本国憲法の文言もまた、国家の権限強化に抑制的である。大日本帝国憲法では国家緊急権が規定されていた一方で、日本国憲法には同様の規定が不在である。参照、高橋和之『立憲主義と日本国憲法〔第 5 版〕』 （有斐閣、2020 年） 、470-471 頁。

^93^
注 64 も参照。

^94^
日本国憲法においては戦争が脅威であるとされ、その結果平和主義が憲法の原則の一つとなっている。

^95^
NHK「外出禁止や休業を強制できる法改正必要 62% NHK 世論調査」 https://www3.nhk.or.jp/news/html/20200623/k10012480041000.html （2020 年 6 月 23 日、2021 年 1 月 1 日アクセス） 。また 2020 年 5 月に JNN が実施した世論調査では、56% が罰則規定の導入に賛成している。 TBS NEWS JNN 世論調査「特措法をめぐる法改正について?」 https://news.tbs.co.jp/newsi_sp/yoron/backnumber/20200509/q2-7.html （2020 年 5 月 9-10 日、2021 年 1 月 10 日アクセス） 。

^96^
NHK「2021 年 1 月 政治意識月例電話調査」 https://www.nhk.or.jp/senkyo/shijiritsu/pdf/aggregate/2021/y202101.pdf （2021 年 1 月 13 日アクセス） 。

^97^
なお、2020 年 11-12 月に朝日新聞が実施した世論調査では、飲食店の休業指示に従わなかった場合の罰金に賛成したのは 33% に留まる。朝日新聞「休業 従わない飲食店に… 『罰金』 33% 『判断尊重』 62% 　本社世論調査」 2021 年 1 月 10 日朝刊 3 面。しかし計 3 回の NHK や JNN による世論調査において半数以上もしくは半数近くが罰則に肯定的な意見を表明しているため、本稿では罰則に肯定的な意見が一定数あると評価した。

^98^
参照、江藤・前掲注 4 、77 頁。

^99^
COVID-19 の影響で新たな国家観が検討されることへの懸念は以下を参照。棟居快行「コロナ禍社会における法的課題(7)コロナの時代の憲法」判例時報 2463 号（2021 年） 87-89 頁。

^100^
勝間靖「新型コロナウイルス感染症 (COVID-19) の感染予防と蔓延防止のための政策 ―ベトナム、タイ、ベルギーでの事例を中心として―」WASEDA ONLINE https://yab.yomiuri.co.jp/adv/wol/opinion/20200430.php （2020 年 4 月 30 日、2021 年 1 月 10 日アクセス） 。

^101^
人間による世界各国での森林開発のため、野生動物のウイルスが人間社会に持ち込まれていることが指摘されている。湯本貴和「コロナ危機は生態系からの警告である」世界 2020 年 8 月 (935) 号 （2020 年） 111-112 頁。

^102^
IPCC, “Summary for Policymakers.” *Climate Change 2021: The Physical Science Basis. Contribution of Working Group I to the Sixth Assessment Report of the Intergovernmental Panel on Climate Change* (V. Masson-Delmotte, P. Zhai, A. Pirani, S. L. Connors, C. Péan, S. Berger, N. Caud, Y. Chen, L. Goldfarb, M. I. Gomis, M. Huang, K. Leitzell, E. Lonnoy, J.B.R. Matthews, T. K. Maycock, T. Waterfield, O. Yelekçi, R. Yu and B. Zhou, Eds). Cambridge University Press, In Press, p. 19. Retrieved from https://www.ipcc.ch/report/ar6/wg1/#SPM (accessed on 16 August 2021).

^103^
しかし、科学的な知見にも政治的な要素が入り得ることには注意が必要である。

^104^
憲法（学）が想定してこなかったリスクとしては、テロリズムなども挙げられる。ただ、テロリズムで想定される「敵」は人間であり、日本国憲法は平和主義に象徴されるように、他国を含めた人間を「敵」とはみなしていない。そのためテロリズムの位置付けは、日本国憲法における平和主義の位置付けにも関わる可能性がある。この論点は、自然環境を「敵」と位置付ける本稿と射程が異なるため、別稿で論じたい。

## データ利用可能性

本論文は法学の論文であり、「生データ (raw data) 」は存在しない。書籍や論文として公開されている学説、判例等に基づいている。

