# Supplementary material for: COVID-19 measures and human rights guaranteed by the Japanese Constitution
Source: F1000Res. 2021 Sep 14;10:230. [Article in Japanese] Originally published 2021 Mar 23. [Version 2] doi: 10.12688/f1000research.50861.2 (PMC8495659; doi:10.12688/f1000research.50861.2)
Supplement: Peer review history

# Peer review: Reviewer response for version 2

**DOI:** 10.5256/f1000research.76767.r94194

**Published:** 2021-09-24

**Authors:** Hajime Yamamoto

**Affiliations:** 1 Keio University, Tokyo, Japan

第1版と比べて、より筆者の立場が明確になったと思われる。筆者の取り上げたテーマは、極めてプラクティカルな問題から、抽象的な憲法解釈の定式化のあり方まで、多様な問題が含まれており、今後はそれらを分節化した上で、さらにそれぞれの論点について考察を進めて欲しい。評者としては、すでに第1版に関して指摘したように、＜しっかりと生命権利益が包含された公共の福祉による移動の自由の制約＞として論点を構成した場合と、＜生命権 vs 移動の自由＞としてそうした場合に、結論を左右するような実質的差異が見出されるのか、両者は表面的な対立に過ぎないのではないか、という疑問について答えるために、憲法解釈論のあり方をなお一般理論のレベルで検討する必要があるように感じられる。

（該当する場合は要回答）統計分析および解釈は適切ですか。

はい

本研究は明確かつ正確に提示されたものであり、最新の文献を引用していますか。

はい

方法と分析について第三者による再現が可能となるよう十分な詳細が提示されていますか。

はい

結果の基礎となるソースデータはすべて入手可能で再現性を十全に保証していますか。

はい

結論は結果により妥当な裏付けを得ていますか。

はい

研究設計は適切で学術的価値がありますか。

はい

Reviewer Expertise:

憲法学

I confirm that I have read this submission and believe that I have an appropriate level of expertise to confirm that it is of an acceptable scientific standard.

# Peer review: Reviewer response for version 2

**DOI:** 10.5256/f1000research.76767.r94193

**Published:** 2021-09-22

**Authors:** Masahiro Sogabe

**Affiliations:** 1 Graduate School of Law, Kyoto University, Kyoto, Japan

第１稿に対するコメントを踏まえ、一定程度の改善はなされたと考える。とりわけ、先行業績への言及や生存権に関する従来の学説に関する指摘には一定の対応がなされている。

 他方で、「個人の自由を保障する概念」対「個人の自由を制限しうる概念」という枠組みでの分析の意義に関する指摘に関しては、なお疑問もあるが、前段落で述べた基本的な点への対応がなされたことを踏まえ、この点については論文公表後、学説での議論に供するのが適当だと考える。

 また、COVID-19対策の諸措置の合憲性の検討については依然として立ち入った論述がないが、これは本論文の主題ではないと理解したため、今後の検討を期待したい。

（該当する場合は要回答）統計分析および解釈は適切ですか。

対象外（統計を使っていない）

本研究は明確かつ正確に提示されたものであり、最新の文献を引用していますか。

一部該当

方法と分析について第三者による再現が可能となるよう十分な詳細が提示されていますか。

はい

結果の基礎となるソースデータはすべて入手可能で再現性を十全に保証していますか。

はい

結論は結果により妥当な裏付けを得ていますか。

はい

研究設計は適切で学術的価値がありますか。

はい

Reviewer Expertise:

憲法

I confirm that I have read this submission and believe that I have an appropriate level of expertise to confirm that it is of an acceptable scientific standard.

# Peer review: Reviewer response for version 1

**DOI:** 10.5256/f1000research.53950.r83082

**Published:** 2021-04-26

**Authors:** Masahiro Sogabe

**Affiliations:** 1 Graduate School of Law, Kyoto University, Kyoto, Japan

本論文は、「COVID-19 対策としての休業や営業時間短縮の要請・指示及び外出自粛要請に、憲法上の制約もしくは要請はあるか」をリサーチクエスチョンとして、憲法学の通説に対する批判も交えつつ、この問いに取り組んでいる。

 本査読者の全般的な評価は、基本的にはすでに査読コメントを残している山元教授と同様であるが、以下の４点について特に指摘しておきたい。

 第１に、筆者は、「公共の福祉」対「個人の人権」という通説的な問題の捉え方に代えて、「個人の自由を保障する概念」対「個人の自由を制限しうる概念」という捉え方を用いるべきだとする。しかし、通説も、「公共の福祉」は抽象的な概念ではなく、具体的な対抗利益を明確にし、その都度の「公共の福祉」の内実を明らかにすべきことを要求しているのであるから、こうした問題設定の修正が何をもたらすのかは明らかではない。

 第２に、筆者は、生存権が具体的な権利であることを強調するが、そこで指摘されている論拠は通説も織り込み済みのものであるはずであり、にもかかわらず通説が抽象的権利説（その内実にも実は注意が必要であるが）にとどまっている理由を乗り越えることができていない。また、本論文の主題との関係で、生存権が具体的権利であるとすることによっていかなる帰結をえようとしているのか、明らかでない。

 第３に、「COVID-19 対策としての休業や営業時間短縮の要請・指示及び外出自粛要請に、憲法上の制約もしくは要請はあるか」というリサーチクエスチョンに対して、一般的な回答を示しているにとどまる。本論文の対象時期においても、法令上の根拠に基づき一定の具体的な対策が展開していたのであるから、それに即した具体論が期待されるところである。

 第４に、冒頭で、COVID-19対策に関して「包括的に検討した」憲法学の論文が少ないとしているが、2020年に限っても、管見の限り下記のようなものが存在する（一部は行政法学者によるものだが。また論文のほか、憲法研究者がメディアの取材に応えた発言も一定数あるだろう）。これらは包括的な検討でないということで考慮されなかった可能性もあるが、絶対数が少ない中、そうした理由で言及をしない理由も乏しいように思われ、先行研究の把握が十分なされたのかどうか不明確である。

 ・大林啓吾「感染症リスクと憲法 新型コロナウイルス流行を素材にして」小山剛、新井誠、横大道聡（編）『日常のなかの〈自由と安全〉生活安全をめぐる法・政策・実務』（弘文堂、2020）410頁

 ・尾形健「新型コロナと法（第5回）「新型コロナウイルス禍」の福祉国家 : 憲法研究者からみた「新型コロナと法」」法学セミナー790号（2020年） 56頁

 ・林知更「法律時評 憲法・非常事態・コロナ」法律時報92巻13号（2020年）1 頁

 ・木村草太「休業・休校要請の法的根拠について 特措法24条9項と45条2項の関係（コロナ禍社会における法的諸問題（３））」判例時報2459号（2020年）157頁

 ・小山剛「自粛・補償・公表 インフォーマルな規制手法（コロナ禍社会における法的諸問題）」判例時報2460号（2020年）145 頁

 ・板垣勝彦「新型コロナウイルス雑感―自粛要請、休業と補償、都市封鎖」横浜法学29巻1号（2020年）185 頁

（該当する場合は要回答）統計分析および解釈は適切ですか。

対象外（統計を使っていない）

本研究は明確かつ正確に提示されたものであり、最新の文献を引用していますか。

一部該当

方法と分析について第三者による再現が可能となるよう十分な詳細が提示されていますか。

はい

結果の基礎となるソースデータはすべて入手可能で再現性を十全に保証していますか。

はい

結論は結果により妥当な裏付けを得ていますか。

はい

研究設計は適切で学術的価値がありますか。

はい

Reviewer Expertise:

憲法

I confirm that I have read this submission and believe that I have an appropriate level of expertise to confirm that it is of an acceptable scientific standard, however I have significant reservations, as outlined above.

# Response

**Published:** 2021-08-19

**Authors:** Hajime Akiyama

**Affiliations:** University of Tsukuba, Japan

曽我部先生

 重要なコメントをいただき、感謝いたします。

 第一に「公共の福祉」と「個人の人権」の分類を、「個人の自由を保障する概念」及び「個人の自由を制限しうる概念」に整理し直す意義についてコメントをいただきました。新たな分類を構想した背景には、「公共の福祉」と「個人の人権」の分類では、人権概念の十全な理解が困難なのではないかとの問題意識があります。人権と自由はほぼ同一視されてきた傾向がありますが、人権を十分に理解するためには、自由権だけでなく、社会権も含めて人権を捉える必要があると考えております。感染症対策との関連では、自由の制限を人権の制約原理と捉えられる公共の福祉でなく、憲法の一義的な価値である人権の一部として位置付けることも可能であるはずです。こうした新たな捉え方により、自由を相対化し、人権のより深い理解を目指したいと考えております。

 第二に、具体的権利説への批判を乗り越えることができていないとのご指摘をいただきました。第2版では、抽象的権利説から具体的権利説に投げかけられている批判を踏まえ、具体的権利説の妥当性を論じました。COVID-19対策との関連では、具体的権利説により、営業や移動の自由の制限が感染対策に有効であれば、こうした措置を採ることが生存権の視点から求められる可能性があると考えています。

 第三に、本論文を基盤とした具体論の展開についてコメントをいただきました。生命権や生存権の具体的権利説を基盤とすれば、感染拡大が止まらない中、科学的知見に依拠したさらに有効な手段による感染拡大の予防が求められると考えております。しかし、2021年2月の特措法改正により導入された罰則の評価など、十分に検討できていない様々な論点がございますので、今後の研究課題としてまいります。

 第四に、先行研究への言及が不十分であるとのご指摘をいただきました。十分に整理できておりませんでしたので、第1版脱稿後に発表された論文も含め勉強させていただき、本論文との関連を検討いたしました。

 上記の論点を踏まえ、第2版を執筆いたしました。

 貴重なコメントを頂き、ありがとうございました。

# Peer review: Reviewer response for version 1

**DOI:** 10.5256/f1000research.53950.r82364

**Published:** 2021-04-07

**Authors:** Hajime Yamamoto

**Affiliations:** 1 Keio University, Tokyo, Japan

本論文は、Covid-19の感染拡大に対応して「新インフルエンザ等対策特別措置法」に基づいて行われた政府による様々な要請や指示を、「憲法の枠組み」で捉えたとき、従来の枠組が不十分だと著者が評価する部分を摘示し、よりよい、と本論文著者が考える憲法学の枠組を提供しようとするものである。

 Covid-19をめぐる問題は、憲法学にとっても最新かつ最重要な諸問題を提起しており、本論文はこのような課題に迅速に応えて執筆されたものである。

 本論文の主張の核心は、憲法の枠組をCovid-19対策で展開されてきた諸措置との関係で捉え直すと、＜個人の人権 vs 公共の福祉＞という二元的対立図式を、憲法上の「個人の自由を保障する概念」と「個人の自由を制限しうる概念」の二つのカテゴリーに再編し、具体的には、個人の人権として位置づけられうる生命権や生存権を、「個人の自由を保障する概念」としてだけではなく、人権制限の契機として作動しうる概念としても位置づけ直すべきだ、とするところにある。こうして具体的には、例えば、Covid-19対策のための諸措置は、生命権保障の手段として位置づけ直されることになる。

 本論文の価値は、著者の考える「憲法の枠組み」がいかなる目的の下に、いかなる役割を担って提出されているか、についての評価によって大きく左右されるのではないか、と考えられる。一方で、著者が位置づけ直そうとする生命権や生存権は、従来の「憲法の枠組み」の中では、生命的価値や公衆衛生的価値としてカウントされており、公共の福祉に吸収されて、殺人罪の処罰や精神病患者の強制入院を憲法上正当化してきた。この点において、本論文の主張は、利益から人権への言葉の言い換えに留まる可能性がある。他方、著者の主張は、例えば、生命権という一つの人権概念が、ある個人の自由権的な権利要求と他者の人権制約をもたらす義務づけ要求を内部に併存させて、一つの人権がベクトルが正反対な多元的な機能を営むものとして再構成させるものである。この点から見ると、人権概念を極めて不明確なもの、あるいは不安定なものとしてしまう、との批判が、従来の憲法学からもたらされる可能性が高い。

 著者の示す実際の政府の諸措置についての合憲性の判定の考え方は、二重の基準や比例原則を援用するオーソドックスな違憲審査の枠組みに落ち着いていて、著者は前半部分で人権概念の大転換を主張しているのにもかかわらず、後半部分における具体的な考察と、従来の憲法学の考え方との違いは、必ずしも大きくない印象がある。

 査読者山元は、従来の近代立憲主義的人権概念を乗り越えようとする著者の基本的スタンスには共感するところが大きい。しかしながら、Covid-19を契機としてそのような試みを行うことは十分理解可能ではあるものの、同じような議論は、例えばテロ対策の文脈でも成り立つように思われる（国民の生命権を保障するための、テロ対策の強化の正当化）。そうだとすると、著者が本論文の問題意識に十分応えるためには、日本の憲法学で歴史的に形成されてきたこれまでのパラダイムの転換という大きな構図を描き、その憲法理論的および実践的意義を説得的に論じる手続がどうしても必要なように思われる。

 関連して、「コロナ自警団」や「自粛警察」の問題は、極めて重要であるが、社会的な制裁が暴力行為に及べば警察による効果的な取り締まりが期待されることは当然であるが、そこには至らない社会的プレッシャーのメカニズムをどのように評価するか、について、著者なりの考察を展開して欲しかった。この点「ゆるやかな規制に対してゆるやかに統制する、ゆるふわ立憲主義」論（曽我部真裕教授）と「市民としての自己拘束」論（江藤祥平教授）が、それぞれ現状の日本を完全に否定しない立場からニュアンスの異なる議論が提出されているところである。著者は、この問題圏についてどのようなアプローチを行うのだろうか。

 こうして、査読者としては、著者のチャレンジングな姿勢を高く評価するとともに、上記の諸論点に関する今後の著者の議論の発展に強く期待したい。

（該当する場合は要回答）統計分析および解釈は適切ですか。

はい

本研究は明確かつ正確に提示されたものであり、最新の文献を引用していますか。

はい

方法と分析について第三者による再現が可能となるよう十分な詳細が提示されていますか。

はい

結果の基礎となるソースデータはすべて入手可能で再現性を十全に保証していますか。

はい

結論は結果により妥当な裏付けを得ていますか。

はい

研究設計は適切で学術的価値がありますか。

はい

Reviewer Expertise:

憲法学

I confirm that I have read this submission and believe that I have an appropriate level of expertise to confirm that it is of an acceptable scientific standard.

# Response

**Published:** 2021-04-18

**Authors:** Hajime Akiyama

**Affiliations:** University of Tsukuba, Japan

山元先生

 有益なご指摘をいただき、感謝申し上げます。

 第一に、生命権や生存権を「個人の自由を制限しうる概念」と捉えることについて、従来「公共の福祉」の利益と考えられてきた概念を「人権」という言葉に言い換えたにすぎないと指摘される可能性をお示しいただきました。筆者は、まさにこの捉え方の変化にこそ大きな意味があると考えております。従来「人権≒自由」と捉えられてきたように思いますが、自由以外の人権の側面を改めて示すことで、山元先生も言及されているように「従来の近代立憲主義的人権概念を乗り越え」る、新たな人権概念を構想する基盤となると存じます。そのため、「個人の自由を制限しうる概念」を「人権」概念に組み込むことには一定の意味があると考えております。

 第二に、人権が個人の自由保障と自由制限双方の正反対な機能を含むと捉えることにより、人権概念が不安定になるとの批判を受ける可能性をご指摘いただきました。筆者は、COVID-19という自然の脅威により、自由を基盤とする従来の人権概念の限界が明らかになっていると考えており、人権の捉え直しが必要であると考えています。そのため、人権概念が不安定になる可能性はありますが、新たな人権概念を検討することで、より包括的な人権概念を構築することができると考えます。

 第三に、前半部分で人権概念の大転換を主張しているのにもかかわらず、後半部分における具体的な考察は、従来の憲法学と大きく変わらないのではないか、とのご指摘をいただきました。本論文の主眼は、日本国憲法の条文を基盤として自由と人権の概念を切り離し、人権概念を捉え直すことにありました。そのため、合憲性判定に関する基準や原則については十分に検討できておりません。人権概念の捉え直しにより、これらの基準や原則を捉え直す必要があるかについては慎重な検討が求められるように思いますが、今後の検討課題とさせていただきます。

 第四に、COVID-19対策とテロ対策は同様に捉えられるか、との論点をご提示いただきました。「おわりに」にも記述しているとおり、本論文は感染症対策における「敵」が、科学的な専門知により認定される自然であると捉えています。従来は、政治的判断が不可避となる人間集団（国家やテロリストを含む）が「敵」として想定されており、COVID-19対策とテロ対策は本質的に異なると考えます。そして、従来想定されてこなかった自然が新たな「敵」となっているため、新たな人権理解の必要性を論じております。テロ対策の捉え方については、今後検討を重ねて参ります。

 第五に、社会的プレッシャーの評価について言及していただきました。この点については、COVID-19対策に法の役割を見出すならば、社会的プレッシャーについては否定的な立場を取らざるを得ません。社会的プレッシャーに法や司法が対応できる余地は限定的だからです。法や司法の意味は、規範が事前に示され、司法による救済の可能性があることだと考えます。社会的プレッシャーについてはこうした法の効果が十分に見込めないため、少なくとも理論的には望ましくありません。とはいえ、曽我部教授、江藤教授の議論にもあるように、日本の社会的現状を踏まえた上で望ましいあり方を模索するのは、極めて重要です。その一方で今後も様々な社会問題が起きる中で、法や司法が十分に役割を果たせる基盤を構築する必要があると感じております。具体的な方法や、その妥当性については大きな論点となりますので、今後の課題とさせてください。

 ご指摘いただきました論点につきましては、今後の論文執筆の参考にさせていただきます。

 貴重なコメントを頂き、ありがとうございました。
